# Suicide Ideation, Attempts, and Mortality in Multiple Sclerosis: A Systematic Review and Meta‐Analysis

**DOI:** 10.1002/brb3.70839

**Published:** 2025-09-09

**Authors:** Omid Mirmosayyeb, Homa Khodadadi, Aynaz Mohammadi, Motahareh Abbasi, Saeed Vaheb, Mohammad Yazdan Panah, Mohammad Mohammadi, Vahid Shaygannejad

**Affiliations:** ^1^ Isfahan Neurosciences Research Center Isfahan University of Medical Sciences Isfahan Iran; ^2^ Department of Neurology Isfahan University of Medical Sciences Isfahan Iran; ^3^ School of Medicine Iran University of Medical Sciences Tehran Iran

**Keywords:** meta‐analysis, mortality, multiple sclerosis, prevalence, suicide

## Abstract

**Background:**

Living with multiple sclerosis (MS) means facing significant obstacles in managing the unpredictable nature of this lifelong condition. Studies highlight a concerning connection between the disease and an elevated risk of suicide. In this study, we assessed the prevalence of suicide and suicide mortality risk in people with MS (PwMS).

**Methods:**

A comprehensive and systematic search of Medline, EMBASE, Scopus, and Web of Science databases was conducted. Studies of any design were included if they reported at least one of the following outcomes: (1) the prevalence of suicide ideation, suicide attempts, suicide deaths, and the proportion of suicide deaths among total deaths in MS populations (2) the risk of suicide mortality in PwMS compared to healthy controls.

**Results:**

The systematic review and meta‐analysis included 64 studies across 19 countries, predominantly from Europe and North America, encompassing over 200,000 PwMS. The pooled prevalence of suicide ideation was 22.6% (95% CI: 16.9–28.3). Suicide attempts were reported at 3.4% (95% CI: 1.6–5.2), while suicide mortality was 0.5% (95% CI: 0.3–0.7), accounting for 2.1% (95% CI: 1.5–2.7) of total mortality in PwMS. PwMS had a significantly higher suicide mortality risk compared to healthy controls (standardized mortality ratio [SMR] = 1.49, 95% CI: 1.08–2.05).

**Conclusion:**

This study highlights the elevated suicide mortality risk among PwMS, underscoring the urgent need for integrated mental health care in MS management. Future research should explore the impact of disease‐modifying therapies, protective factors, and standardized risk assessment tools to improve early intervention and reduce suicidal behavior in this vulnerable population.

## Graphical abstract

This systematic review and meta‐analysis of 64 studies including over 200,000 people with multiple sclerosis (PwMS) revealed a high prevalence of suicide ideation (22.6%), attempts (3.4%), and suicide mortality (2.1%) in PwMS. This study highlights the elevated suicide mortality risk among PwMS, underscoring the need for integrated mental health care in MS management.

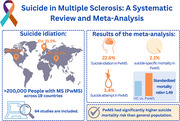

## 1 Introduction

Multiple sclerosis, (MS) as a long‐term inflammatory condition, affects the central nervous system (CNS). Globally, it impacts an estimated 2.5 to 2.8 million individuals (Pompili et al. 2012, Walton et al. 2020), leading to a variety of challenging physical, cognitive, and emotional symptoms that are often difficult to manage and can lead to disability. Living with MS means facing significant obstacles in managing the unpredictable nature of this lifelong condition (Inojosa et al. 2021). People with MS (PwMS) also frequently experience depression, and studies highlight a concerning connection between the disease and an elevated risk of suicide (Raimo et al. 2021).

Research indicates that suicide prevalence is notably higher among PwMS, as shown in multiple studies (Pompili et al. 2012, Bolton et al. 2015, Brenner et al. 2016, Brønnum‐Hansen et al. 2004, Brønnum‐Hansen et al. 2005, Capkun et al. 2015, Fredrikson et al. 2003, Koch‐Henriksen et al. 1998, Lunde et al. 2017, Sadovnick et al. 1991, Scalfari et al. 2013, Stenager et al. 1992, Kingwell et al. 2020). However, other studies suggest that suicide prevalence in PwMS may be comparable to or even lower than that in the general population (Goodin et al. 2014, Hirst et al. 2008, Kalson‐Ray et al. 2017, Lalmohamed et al. 2012, Marrie et al. 2018, Sandi et al. 2016, Smestad et al. 2009, Sumelahti et al. 2010, Willumsen et al. 2022). These conflicting findings may stem from variations in study design, sample sizes, geographic locations, or patient demographics.

Two recent meta‐analyses have contributed important insights into suicide‐related outcomes in PwMS, but both have notable limitations. Shen et al. (2019) included 16 studies published up to December 2018 and focused exclusively on suicide mortality in PwMS compared to the general population (Shen et al. 2019). Using suicide rate ratio (SRR) as the sole effect size, they reported a significantly elevated risk of suicide (pooled SRR = 1.72), with the highest risk occurring around the time of diagnosis (SRR = 2.12) compared to symptom onset (SRR = 1.69) (Shen et al. 2019). However, this analysis did not evaluate suicide ideation or suicide attempts, which are critical early indicators of suicide risk, and relied solely on SRR without incorporating other commonly used metrics such as odds ratios (OR) or standardized mortality ratios (SMR). A separate meta‐analysis by Kouchaki et al. (2020) estimated the global prevalence of suicide ideation in PwMS at 25.7% based on nine studies, reinforcing the significance of psychological distress in this population (Kouchaki et al. 2020). Nevertheless, their study also had a narrow focus, excluding suicide attempts, mortality data, and comparisons with healthy controls. Given these limitations and the number of additional studies published in recent years, a comprehensive and updated synthesis is warranted. This systematic review and meta‐analysis was conducted to address this critical gap.

Our goal in this study is to comprehensively investigate the association between MS and suicide. Specifically, we assessed the prevalence of suicide ideation, suicide attempts, suicide deaths, and the proportion of suicide deaths among total deaths in MS populations. We will also calculate key measures to compare suicide mortality in PwMS and healthy controls. By synthesizing and analyzing data from multiple studies, this review attempts to provide evidence‐based insights that can improve clinical practices, enhance mental health support for PwMS, and help develop targeted interventions to reduce suicide risk in this vulnerable population.

## 2 Materials and Methods

### 2.1 Search

Two independent reviewers (H.Kh. and M.A.) searched the Medline, Scopus, EMBASE, and Web of Science databases systematically up to the 22^nd^ of June 2024. The Preferred Reporting Items for Systematic Reviews and Meta‐Analyses (PRISMA) checklist's recommendations were followed in this systematic review and meta‐analysis (Page et al. 2021). The detailed search strategy is available in the supplementary materials (Supplementary materials 1, Table S1). The PROSPERO registration number for this systematic review and meta‐analysis is CRD42024628244.

### 2.2 Eligibility Criteria

Studies of any design that reported one or more of the following outcomes were included: (1) the prevalence of suicide ideation, suicide attempts, suicide deaths, and the proportion of suicide deaths among total deaths in MS populations; and (2) the risk of suicide mortality in PwMS compared to healthy controls. Studies were eligible if they provided any form of the effect size related to the risk of suicide mortality (SMR, OR, HR [hazard ratio], MRR [mortality rate ratio]) or if they reported sufficient data to allow for the calculation of such risk measures.

Review articles, preprinted articles, animal studies, case reports, and case series were excluded. Additionally, studies that focused on other demyelinating conditions, such as clinically isolated syndrome (CIS) and neuromyelitis optica spectrum disorder (NMOSD), were excluded due to substantial differences in their underlying pathology, disease course, and potentially distinct psychiatric and suicide risk profiles compared to MS.

### 2.3 Study Selection and Data Extraction

The initial screening of titles and abstracts was conducted by two reviewers (H.Kh. and M.A.) independently. Then, the same reviewers screened the full‐texts of potentially relevant articles. Furthermore, a screening of the included studies' reference lists was conducted to find any possibly relevant articles. After identifying studies that fulfilled the eligibility criteria through this screening process, two reviewers (H.Kh. and M.A.) independently extracted relevant data. Data such as the first author's name, the study design, sample size, Expanded Disability Status Scale (EDSS), the year of publication, the country, the MS type of PwMS, the disease duration, and the suicide assessment tool used in each study were extracted for each included study. In cases where a study reported prevalence at multiple time points, we extracted data from the most recent or the most representative time point to avoid duplication in pooled analyses.

### 2.4 Risk of Bias Assessment

We assessed the quality of the studies included in the systematic review using the Newcastle‐Ottawa Scale (NOS) (Wells et al. 2000). This scale evaluates important aspects like exposure assessment, case‐control group comparability, and sample selection. It is specifically designed for observational investigations. Higher NOS ratings indicate higher‐quality studies. The values range from 0 to 9. We divided the research into three quality groups based on these scores: studies with 7‐9 stars were classed as high quality, those with 4‐6 stars as moderate quality, and those with less than four stars as low quality.

Two authors (S.V. and M.Y.) independently conducted the quality assessment of the included studies. Most of the studies had moderate to high quality. The scores are available in Table 1.

**TABLE 1:** Fundamental characteristics of included studies.

First author [Ref]	Country year	Study design	PwMS				HCs	Suicide assessment tool	Key findings	QA
			Sample size F:M Age; years: mean (SD)	EDSS	Disease duration; years: mean (SD)	MS type	Sample size F:M Age; years: mean (SD)			
Mikula et al. 2024	Slovakia 2024	Cross sectional	162 123:39 40.9 (11.4)	3.5 (1.1)	11.6 (7.1)	NR	NR	GHQ‐28	SI prevalence was higher in PwMS compared to the HCs.	7
Barak‐Corren et al. 2023	US 2023	Cohort	15117 11072:4043 54.6	NR	NR	NR	14920 10939:3979 54.5	ICD‐9	Suicidal behaviors have increased among PwMS.	9
Eliasdottir et al. 2023	Sweden 2023	Cohort	526 382:144 NR	NR	NR	RRMS: 386 PPMS:35	NR	Death certificates	The mortality of suicide in PwMS and the HSc are same.	6
Willumsen et al. 2022	Norway 2022	Cohort	964 535:429 NR	NR	4.3	RRMS: 715 PPMS: 237 Unknown: 12	NR	Death certificates	The mortality of suicide in PwMS and the HSc are same.	8
Schorr et al. 2022	US 2022	Cohort	7698 5596:2102 45.4 (13.9)	NR	NR	NR	NR	ICD‐9	There is no significant association between readmission due to SA and hospitalization for MS.	9
Sariaslani et al. 2021	Iran 2021	Cross sectional	203 157:46 36.2 (10.2)	NR	NR	NR	NR	BSS	The prevalence of SI and SA are significantly higher in PwMS compared to HCs.	7
Murtonen et al. 2021	Finland 2021	Cohort	113 63:50 NR	NR	16.5 (8.5)	RRMS:90 PPMS:23	NR	Death certificates	Suicide is a leading cause of death among young PwMS.	7
Pouradeli et al. 2024	Iran 2021	Cross sectional	202 178:24 38 (9.1)	NR	7.3 (6.4)	NR	NR	BSSI	Low percentage of PwMS was at high risk for suicide.	6
Văcăraș et al. 2020	Romania 2020	Cross sectional	146 105:41 40.8 (11.5)	2.3 (0 − 6.5)*	6 (6.3)	RRMS:128 SPMS:10 CIS:8	NR	BDI‐II	A low SI and a low percentage of deaths by suicide are observed in PwMS.	7
Erlangsen et al. 2020	Denmark 2020	Cohort	31136 NR NR	NR	NR	NR	7233928 NR NR	ICD‐8	There is a significant link between frequent hospital visits among PwMS and suicide.	8
Knowles et al. 2020	US 2020	Cross sectional	573 470:103 54.4 (10.8)	NR	15.3 (10.1)	PMS:142 RRMS:431	NR	PHQ‐9	PwMS with more severe MS‐related impairments are at greater risk for suicide.	10
Romaniuc et al. 2020	Romania 2020	Cross sectional	349 237:112 42	2.5 (0 − 8.5)*	10	RRMS:245 SPMS:94 CIS:10	NR	BDI‐II	A positive correlation was observed between the severity of the SI and EDSS.	8
Asgarian et al. 2020	Iran 2020	Cross sectional	276 205:71 35.8 (8.6)	2.6 (1.7)	6.3 (5.8)	RRM:199 PPMS:31 SPMS:35 PRMS:11	NR	PHQ‐9	A significant percentage of PwMS had SI.	6
Kingwell et al. 2020	Canada 2019	Cohort	6629 4807:1823 63 (53 − 73)**	NR	26 (18 − 36)**	NR	NR	ICD	Suicide is one of the significant causes of death among the PwMS.	9
Marrie et al. 2018	Canada 2018	Cohort	27354 19465:7889 NR	NR	13.4 (6.4 − 22.6)**	NR	NR	Death certificates	Suicide is one of the significant causes of death among the PwMS.	6
Eliasen et al. 2018	Germany 2018	Case‐control	48 NR 47.9 (13.3)	NR	NR	NR	314 NR 47 (12.6)	ICD‐10	The risk of suicide in PwMS is higher than in the HCs.	7
Fernández et al. 2018	Argentina 2018	Cohort	18 11:7 44.7 (27 − 61)^z^	3 (1 − 7)^z^	11.7 (1 − 35)^z^	NR	20 14:6 42 (25 − 65)^z^	BDI SCID	Risk of suicide was moderate in PwMS.	4
Tauil et al. 2018	Brazil 2018	Cross sectional	132 103:29 35 (18 − 65)^z^	2.5 (0 − 7.5)^z^	NR	RRMS:132	NR	BDI‐II	There is an Increased risk of SI in PwMS.	6
Kalson‐Ray et al. 2017	France 2017	Cohort	27603 NR NR	4.6 (2.2)	11 (10.3)	NR	NR	ICD‐10	There is no increased risk of SA among PwMS versus the HCs.	7
Burkill et al. 2017	Sweden 2017	Case‐control	29617 19658:9959 NR	NR	NR	NR	296164 196576:99588 NR	Death certificates	There was no significant increase in suicide among the PwMS.	7
Lunde et al. 2017	Norway 2017	Cohort	291 160:131 NR	NR	NR	NR	NR	Death certificates	There was no significant increase in suicide among the PwMS.	7
Brenner et al. 2016	Sweden 2016	Case‐control	29164 NR NR	NR	NR	NR	288151 NR NR	Death certificates	There was a significant increase in suicide among the PwMS.	7
Lewis et al. 2017	UK 2016	Cross sectional	75 44:31 55 (10.8)	NR	23 (12.4)	NR	NR	BSS	There is a positive association between disability and SI in PwMS.	7
Sandi et al. 2016	Hungary 2016	Cohort	740 536:204 NR	3.3	NR	RRMS + SPMS:688 PPMS:52	NR	Data base	Suicide rates in PwMS are similar to the HCs.	8
Alamri and Al‐Busaidi 2016	Saudi Arabia 2016	Cross sectional	24 15:9 NR	NR	NR	NR	NR	HADS	A high incidence of SI was found in PwMS.	4
Altura et al. 2016	Canada 2016	Cohort	150 115:35 49.9 (24.9 − 82.3)^z^	NR	NR	NR	NR	PHQ‐9	The prevalence of SI in PwMS is higher than the HCs.	6
Ariapooran et al. 2016	Iran 2016	Cross sectional	79 44:35 37.3 (9.4)	NR	6.1 (4.6)	NR	NR	BSS	A high prevalence of SI was detected in PwMS.	5
Ernst et al. 2016	US 2016	Case‐control	1518 1066:452 NR	NR	NR	NR	305339 171210:134129 NR	Death certificates	There was no significant increase in suicide among the PwMS.	8
Leray et al. 2015	France 2015	Cohort	1569 884:685 56 (11.4)	NR	20 (12.9)	NR	NR	Death certificates	There was no significant increase in suicide among the PwMS.	8
Capkun et al. 2015	US 2015	Case‐control	15684 11992:3692 46 (11.7)	NR	NR	NR	78420 59960:18460 46 (11.7)	Death certificates	The risk of suicide is high in the MS community.	8
Bolton et al. 2015	Canada 2015	Cross sectional	NR	NR	NR	NR	NR	Death certificates	There was a significant increase in suicide among the PwMS.	6
De Cerqueira et al. 2015	Brazil 2015	Case‐control	20 14:6 43.7 (9.8)	NR	NR	NR	NR	MINI	There is a high risk of suicide in PwMS, who have SI.	5
De Cerqueira et al. 2015	Brazil 2015	Cross sectional	60 46:14 43 (11.8)	NR	9.6 (6.8)	RRMS:49 NR:11	NR	BDI BAI SCID	The risk of suicide is high in the MS community.	6
Jick et al. 2014	US 2014	Case‐control	1822 1341:481 42.1 (11.8)	NR	NR	RRMS: 769 PPMS: 125 NR: 928	18211 NR NR	Death certificates	There was no significant increase in suicide among the PwMS.	8
Manouchehrinia et al. 2014	UK 2014	Cross sectional	80 34:46 56 (12)	7.5 (6.5‐8)	18 (10‐26)	RRMS: 9 PPMS: 16 SPMS: 55	NR	Death certificates	There was no significant increase in suicide among the PwMS.	6
Dickstein et al. 2015	US 2014	Cross sectional	3823 2867:956 51 (12)	NR	NR	NR	NR	PHQ‐9	The risk of SI in PwMS is higher compared to HCs.	7
Rodriguez‐Antiguedad Zarranz et al. 2014	Spain 2014	Cohort	1283 NR	NR	12.8 (9.8)	NR	NR	Death certificates	There was no significant increase in suicide among the PwMS.	7
Goodin et al. 2014	US 2014	Cohort	30402 23364:7038 44 (10.8)	NR	NR	NR	89818 69102: 20716 44 (10.8)	Death certificates	Death from suicide was not significantly different between the PwMS and HCs.	8
Viner et al. 2014	Canada 2014	Cross sectional	630 462:268 51.8	NR	NR	NR	NR	PHQ‐9	There are significantly high SI among PwMS.	5
Flood et al. 2014	US 2013	Cohort	344 249:95 NR	NR	NR	NRcohort	NR	BDI‐II	There are significantly high SI among PwMS.	6
Lalmohamed et al. 2012	Netherland 2012	Cohort	1270 899:371 45.6 (13.9)	NR	NR	NR	7648 5418:2230 45.6 (13.8)	ICD‐10	Death by suicide amongst PwMS did not significantly differ from the HCs.	8
Goodin et al. 2012	US 2012	RCT	366 NR 56.3 (7.1)	NR	NR	RRMS:366	NR	Death certificates	Suicide is considered an MS‐related cause of death.	—
Askey‐Jones et al. 2012	UK 2012	Cross sectional	90 65:25 43 (11.4)	NR	8.6 (7.3)	NR	NR	HADS BAI BDI‐II	Gender and type of MS are important risk factors for SI in PwMS.	7
Stenager et al. 2011	Denmark 2011	Cohort	404 283:121 NR	NR	NR	NR	NR	RSA	PwMS did not find an increased risk of SA.	7
Sumelahti et al. 2010	Finland 2010	Cohort	1595 1045:550 NR	NR	NR	RRMS: 1069 PPMS: 335 Unknown: 191	NR	ICD‐8 ICD‐9 ICD‐10	MS disease progression increases suicide risk.	8
Smestad et al. 2009	Norway 2009	Cohort	386 236:150 62.7 (11.7)	NR	30	RRMS:284 PPMS:93 NR:9	NR	ICD	Suicide risk in untreated PwMS was significantly high.	7
Redelings et al. 2006	US 2005	Cohort	44637 28815:15822 NR	NR	NR	NR	NR	Death certificates	—	6
Riudavets et al. 2005	US 2005	Cross sectional	50 32:18 45.8 (25 − 69)^z^	NR	NR	NR	NR	Death certificates	Suicide is the third most prevalent cause of death among PwMS.	7
Patten et al. 2005	Canada 2005	RCT	4469 NR NR	NR	NR	RRMS:3450 SPMS:1019	NR	Death certificates	Suicide is considered one of the causes of death among PwMS.	—
Bronnum‐Hansen et al. 2005	Denmark 2005	Cohort	10174 6113:4061 NR	NR	NR	NR	NR	Death certificates	Suicide risk among PwMS is higher for men than women.	8
Brønnum‐Hansen et al. 2004	Denmark 2004	Cross sectional	9881 5927:3954 NR	NR	NR	NR	NR	Death certificates	The study found a high suicide risk among MS patients.	7
Quesnel and Feinstein 2004	Canada 2004	Cross sectional	140 104:56 43.9 (10.8)	3.6 (2.5)	8.8 (6.8)	NR	NR	BSS	PwMS have a higher prevalence of SI compared to the HCs.	7
Fredrikson et al. 2003	Sweden 2003	Cohort	12834 7984:4850 NR	NR	NR	NR	NR	Death certificates	The study found a high suicide risk among MS patients.	7
Feinstein 2002	Canada 2002	Cross sectional	140 104:36 43.9 (10.7)	3.6 (2.5)	8.8 (6.8)	RRMS:74 NR:66	NR	BSS	The SA in PwMS is higher than in the HCs.	6
Ford et al. 2002	UK 2002	Cross sectional	57 NR NR	NR	NR	NR	NR	Death certificates	The study found a high suicide risk among MS patients.	4
Feinstein et al. 1999	Canada 1999	Cross sectional	152 107:45 45.4 (11.2)	4.9 (2.5)	9.4 (6.9)	NR	NR	HADS	Anxiety and depression contribute significantly to SI in PwMS.	6
Berkman et al. 1999	US 1999	Cross sectional	505 395:98 50.4 (12)	NR	NR	NR	NR	Database	PwMS had contemplated assisted suicide more than the HCs.	6
Salmaggi et al. 1998	Italy 1998	Cross sectional	65 40:25 34 (15 − 63)^z^	4.5 (0 − 8.5)	7 (0 − 32)^z^	RRMS:32 PPMS:11 SPMS:22	NR	DRS BDI	SA were more common in PwMS than HCs.	5
Fisk et al. 1998	Canada 1998	Case‐control	708 NR	NR	NR	NR	NR	ICD‐9	The estimated rate of SA was at least three times higher in hospitalized PwMS than HCs.	4
Koch‐Henriksen et al. 1998	Denmark 1998	Cross sectional	6068 NR NR	NR	NR	NR	NR	Death certificates	An increased risk of death from suicide and accidents can be indirectly attributed to MS.	7
Stenager et al. 1996	Denmark 1996	Cohort	5525 NR NR	NR	NR	NR	NR	Death certificates	Number of SA and deaths by suicide is significantly higher in PwMS.	5
Stenager et al. 1992	Denmark 1992	Cohort	5525 3191:2334 NR	NR	NR	NR	NR	BDI	A small percentage of PwMS have died by suicide.	5
Sadovnick et al. 1991	Canada 1991	Cross sectional	3126 NR NR	NR	NR	NR	NR	Death certificates	The study reveals a significantly higher rate of suicide among PwMS.	5
Eklund and Macdonald 1991	US 1991	Cross sectional	125 94:30 48.6 (12.2)	NR	8.4 (6.9)	NR	NR	BSS	There is an increased suicidality in PwMS.	6

*: Median (range), **: Median (IQR), ^Z^: Mean (range)Abbreviations: RRMS, relapsing remitting MS;PwMS, patients with multiple sclerosis; SPMS, secondary progressive MS; PPMS, primary progressive; PRMS, progressive relapsing MS; BDI‐II, beck depression inventory‐II; BSS, Beck scale for suicide; HADS, hospital anxiety and depression scale; SCID‐IV, clinical interview for DSM‐IV disorders; BAI, beck anxiety inventory; PHQ‐9, patient health questionnaire; BSSI, Beck scale for suicidal ideation; SA, suicide attempt; EDSS, expanded disability status scale; DRS, depression rating scale; MINI, mini‐international neuropsychiatric interview; RSA, Danish register for suicide attempts;, GHQ‐28, general health questionnaire‐28; SI, suicide ideation; HCs, healthy control; PwMS, people with MS; RCT, randomize control trial.

### 2.5 Statistical Analysis

For all analyses, R version 4.4.0 (R Project for Statistical Computing) (Team 2010) with the meta package (Balduzzi et al. 2019) was utilized. The threshold for statistical significance was a two‐sided *p*‐value of less than 0.05. The primary outcomes of meta‐analyses include prevalence, OR, HR, MRR, and SMR. Quantitative analyses of the included studies were conducted using the metaprop function for pooled prevalence, the metabin function for pooled ORs, and the metagen function for pooled HR, SMR, and MRR. The pooled effect estimates, along with their corresponding 95% confidence intervals (CI), were reported. A minimum of three common effect sizes from the included studies was required to conduct each meta‐analysis. To identify potential sources of statistical heterogeneity, we conducted post‐hoc subgroup analyses and meta‐regressions. Subgroup analyses were conducted based on sample size, age, continent, and suicide ideation assessment tool, where applicable, to identify the source of heterogeneity. For the assessment of suicide ideation, the Beck Scale for Suicidal Ideation (BSSI) was used as the suicide ideation‐specific instrument among included studies, while the other questionnaires were considered general screening tools for evaluating suicide ideation. The cutoff of continuous variables in subgroup analyses was selected post‐hoc based on the distribution of their mean reported in included studies and common thresholds used in the MS literature. A random‐effect model was used to calculate pooled outcome measures and their associated 95% CI in order to account for the possible methodological interstudy heterogeneity. The Cochran's Q test and the inconsistency index (I^2^) were utilized to assess statistical heterogeneity among the included studies (Hardy and Thompson 1998). To determine the sensitivity of our aggregated research outcomes, we sequentially excluded individual studies and re‐conducted the pooled analysis (Lau et al. 2006). Publication bias was assessed through visual inspection of the funnel plot, along with statistical evaluations using Begg's and Egger's tests (Begg and Mazumdar 1994, Egger et al. 1997).

## 3 Results

### 3.1 Study Selection and Characteristics

**FIGURE 1:**
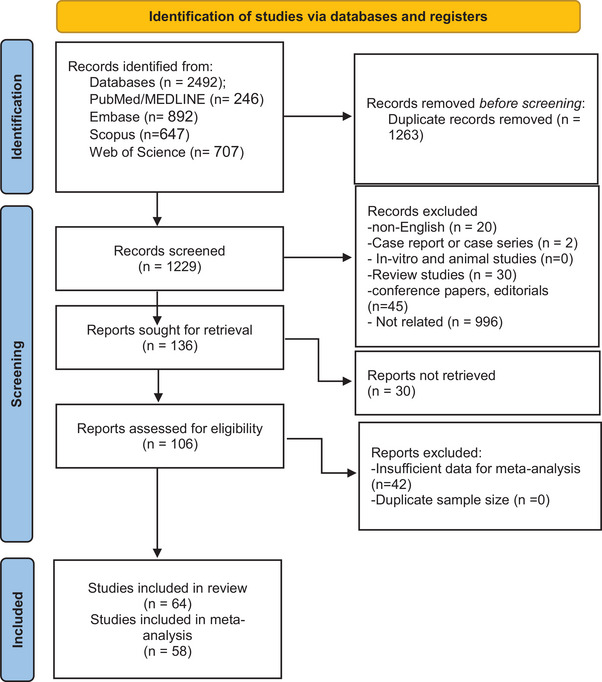
PRISMA diagram of the study selection.

Our search yielded 1263 records, which resulted in 1229 citations after duplicate removal. After primary screening based on title and abstract, 1123 studies were excluded, and the remaining 106 were screened by their full texts. Finally, a total of 64 studies (Bolton et al. 2015, Brenner et al. 2016, Brønnum‐Hansen et al. 2004, Brønnum‐Hansen et al. 2005, Capkun et al. 2015, Fredrikson et al. 2003, Koch‐Henriksen et al. 1998, Lunde et al. 2017, Sadovnick et al. 1991, Stenager et al. 1992, Kingwell et al. 2020, Goodin et al. 2014, Kalson‐Ray et al. 2017, Lalmohamed et al. 2012, Marrie et al. 2018, Sandi et al. 2016, Smestad et al. 2009, Sumelahti et al. 2010, Willumsen et al. 2022, Mikula et al. 2024, Barak‐Corren et al. 2023, Eliasdottir et al. 2023, Schorr et al. 2022, Sariaslani et al. 2021, Murtonen et al. 2021, Pouradeli et al. 2024, Văcăraș et al. 2020, Erlangsen et al. 2020, Knowles et al. 2021, Romaniuc et al. 2020, Asgarian et al. 2020, Eliasen et al. 2018, Fernández et al. 2018, Tauil et al. 2018, Burkill et al. 2017, Lewis et al. 2017, Alamri and Al‐Busaidi 2016, Altura et al. 2016, Ariapooran et al. 2016, Ernst et al. 2016, Leray et al. 2015, De Cerqueira et al. 2015, Jick et al. 2014, Manouchehrinia et al. 2014, Dickstein et al. 2015, Rodríguez‐Antigüedad Zarranz et al. 2014, Flood et al. 2014, Goodin et al. 2012, Askey‐Jones et al. 2012, Cerqueira et al. 2015, Stenager et al. 2011, Redelings et al. 2006, Riudavets et al. 2005, Patten et al. 2005, Quesnel and Feinstein 2004, Feinstein 2002, Ford et al. 2002, Feinstein et al. 1999, Berkman et al. 1999, Salmaggi et al. 1998, Fisk et al. 1998, Stenager et al. 1996, Eklund and MacDonald 1991, Viner et al. 2014) were included in this systematic review and meta‐analysis, encompassing a diverse range of geographic regions, study designs, and sample sizes (Figure 1). The studies were conducted across 19 countries, most of them from Europe (n = 30) (Brenner et al. 2016, Brønnum‐Hansen et al. 2004, Brønnum‐Hansen et al. 2005, Fredrikson et al. 2003, Koch‐Henriksen et al. 1998, Lunde et al. 2017, Stenager et al. 1992, Kalson‐Ray et al. 2017, Lalmohamed et al. 2012, Sandi et al. 2016, Smestad et al. 2009, Sumelahti et al. 2010, Willumsen et al. 2022, Mikula et al. 2024, Eliasdottir et al. 2023, Murtonen et al. 2021, Văcăraș et al., Erlangsen et al. 2020, Romaniuc et al. 2020, Eliasen et al. 2018, Burkill et al. 2017, Lewis et al. 2017, Leray et al. 2015, Manouchehrinia et al. 2014, Rodríguez‐Antigüedad Zarranz et al. 2014, Askey‐Jones et al. 2012, Stenager et al. 2011, Ford et al. 2002, Salmaggi et al. 1998, Stenager et al. 1996) and North America (n = 25) (Bolton et al. 2015, Capkun et al. 2015, Sadovnick et al. 1991, Kingwell et al. 2020, Goodin et al. 2014, Marrie et al. 2018, Barak‐Corren et al. 2023, Schorr et al. 2022, Knowles et al. 2021, Altura et al. 2016, Ernst et al. 2016, Jick et al. 2014, Dickstein et al. 2015, Flood et al. 2014, Goodin et al. 2012, Redelings et al. 2006, Riudavets et al. 2005, Patten et al. 2005, Quesnel and Feinstein 2004, Feinstein 2002, Feinstein et al. 1999, Berkman et al. 1999, Fisk et al. 1998, Eklund and MacDonald 1991, Viner et al. 2014), and others from Asia (n = 5) (Sariaslani et al. 2021, Pouradeli et al. 2024, Asgarian et al. 2020, Alamri and Al‐Busaidi 2016, Ariapooran et al. 2016) and South America (n = 4) (Fernández et al. 2018, Tauil et al. 2018, De Cerqueira et al. 2015, Cerqueira et al. 2015). The publication years of the included studies ranged from 1991 to 2024, with the majority published in the last decade (2010–2024).

The study designs varied, including cross‐sectional studies (n = 28) (Bolton et al. 2015, Brønnum‐Hansen et al. 2004, Koch‐Henriksen et al. 1998, Sadovnick et al. 1991, Mikula et al. 2024, Sariaslani et al. 2021, Pouradeli et al. 2024, Văcăraș et al. 2020, Knowles et al. 2021, Romaniuc et al. 2020, Asgarian et al. 2020, Tauil et al. 2018, Lewis et al. 2017, Alamri and Al‐Busaidi 2016, Ariapooran et al. 2016, De Cerqueira et al. 2015, Manouchehrinia et al. 2014, Dickstein et al. 2015, Askey‐Jones et al. 2012, Riudavets et al. 2005, Quesnel and Feinstein 2004, Feinstein 2002, Ford et al. 2002, Feinstein et al. 1999, Berkman et al. 1999, Salmaggi et al. 1998, Eklund and MacDonald 1991, Viner et al. 2014), cohort studies (n = 26) (Brønnum‐Hansen et al. 2005, Fredrikson et al. 2003, Lunde et al. 2017, Stenager et al. 1992, Kingwell et al. 2020, Goodin et al. 2014, Kalson‐Ray et al. 2017, Lalmohamed et al. 2012, Marrie et al. 2018, Sandi et al. 2016, Smestad et al. 2009, Sumelahti et al. 2010, Willumsen et al. 2022, Barak‐Corren et al. 2023, Eliasdottir et al. 2023, Schorr et al. 2022, Murtonen et al. 2021, Erlangsen et al. 2020, Fernández et al. 2018, Altura et al. 2016, Leray et al. 2015, Rodríguez‐Antigüedad Zarranz et al. 2014, Flood et al. 2014, Stenager et al. 2011, Redelings et al. 2006, Stenager et al. 1996), case‐control studies (n = 8) (Brenner et al. 2016, Capkun et al. 2015, Eliasen et al. 2018, Burkill et al. 2017, Ernst et al. 2016, Jick et al. 2014, Cerqueira et al. 2015, Fisk et al. 1998), and randomized controlled trials (n = 2) (Goodin et al. 2012, Patten et al. 2005). The sample sizes of PwMS ranged from 18 to 31,136, with a total pooled sample size of > 200,000 PwMS across all studies. Healthy controls were included in 18 studies, with sample sizes ranging from 20 to 7,233,928.

The PwMS participants' average age was between 34 and 63 years old, and the ratio of women to men was consistently skewed in favor of women. The mean sample size across the included studies was approximately 5300 PwMS, with a range from 18 to 44637 participants. The mean duration of the disease ranged from 4.3 to 30 years, and the EDSS scores, where reported, ranged from 2.3 to 7.5. The types of MS reported included relapsing‐remitting MS (RRMS), primary progressive MS (PPMS), secondary progressive MS (SPMS), and progressive relapsing MS (PRMS), with RRMS being the most frequently reported MS type. Studies’ detailed characteristics are available in Table 1.

### 3.2 Prevalence of Suicide‐Related Outcomes in PwMS

#### 3.2.1 Suicide Ideation

**FIGURE 2:**
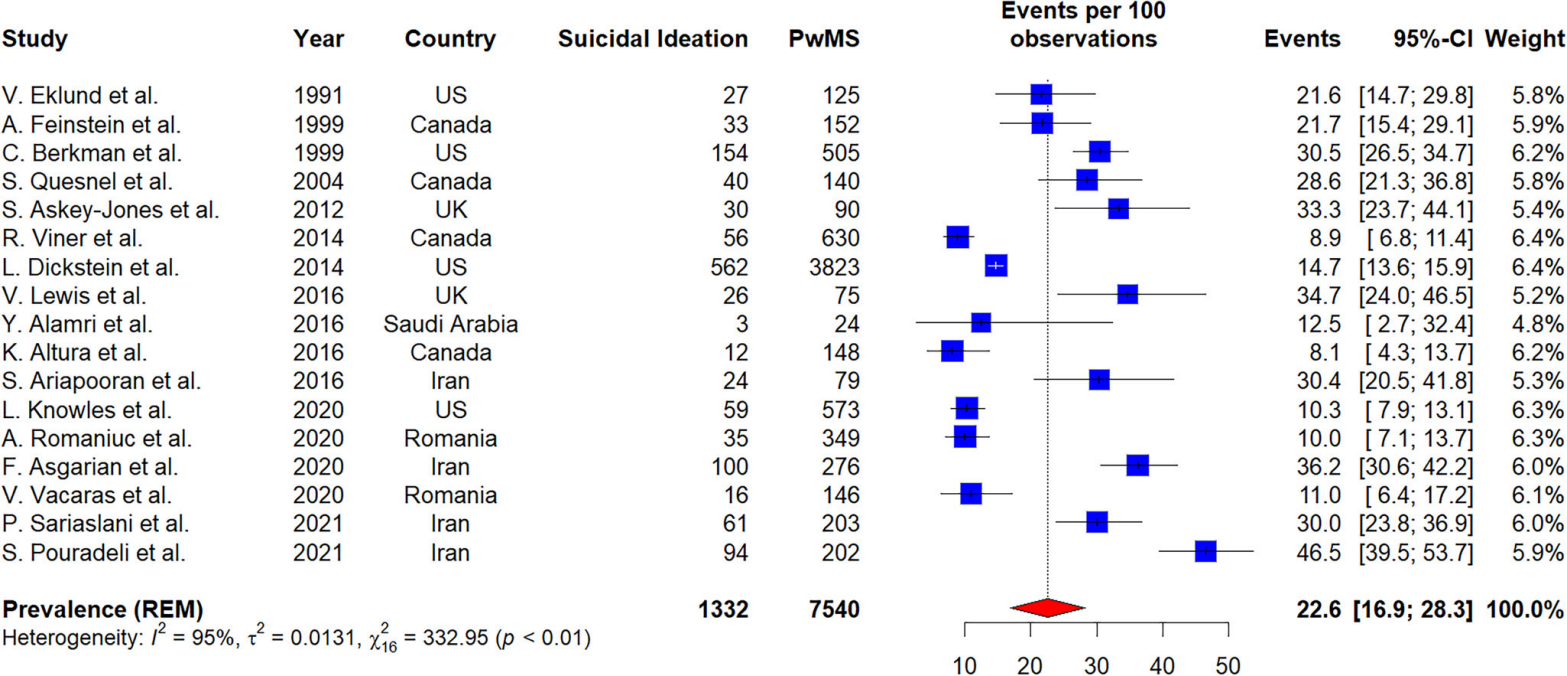
Prevalence of suicide ideation in PwMS.

In PwMS, the pooled prevalence of suicide ideation was 22.6% (number of studies = 17, 95% CI: 16.9–28.3, I^2^ = 95%, p heterogeneity < 0.01) (Figure 2). A prevalence of 21.5% (95% CI: 8.2–34.8, I^2^ = 92%, *p*‐value for heterogeneity < 0.01) was reported by studies in Europe, 17.7% (95% CI: 11.6–23.9, I^2^ = 94%, *p*‐value for heterogeneity < 0.01) by studies in America, and 32.0% (95% CI: 21.9–42.1, I^2^ = 84%, *p*‐value for heterogeneity < 0.01) by studies in Asia. The overall test for subgroup differences between geographic subgroups was not statistically significant (*p*‐value for subgroup difference = 0.06). According to age subgroup analysis, PwMS under 45 had a greater prevalence of suicide ideation (28%, 95% CI: 19.2–36.8, I^2^ = 96%, *p*‐value for heterogeneity < 0.01) than PwMS over 45 (17.8%, 95% CI: 10.2–25.4, I^2^ = 95%, p heterogeneity < 0.01) although the difference was not statistically significant (*p*‐value for subgroup difference = 0.09). Subgroup analysis based on the suicide ideation assessment tools indicated that the pooled prevalence of suicide ideation among studies that used suicide ideation‐specific instruments (31.7%, 95% CI: 25.9–37.5, I^2^ = 77.8%, *p*‐value for heterogeneity < 0.01) was significantly higher than in studies that used general screening instruments (16.3%, 95% CI: 10.1–22.6, I^2^ = 92.6%, *p*‐value for heterogeneity < 0.01) (*p*‐value for subgroup difference < 0.01). Additionally, meta‐regression revealed no significant association between the prevalence of suicide ideation in PwMS and either age or disease duration.

#### 3.2.2 Suicide Attempt

**FIGURE 3:**
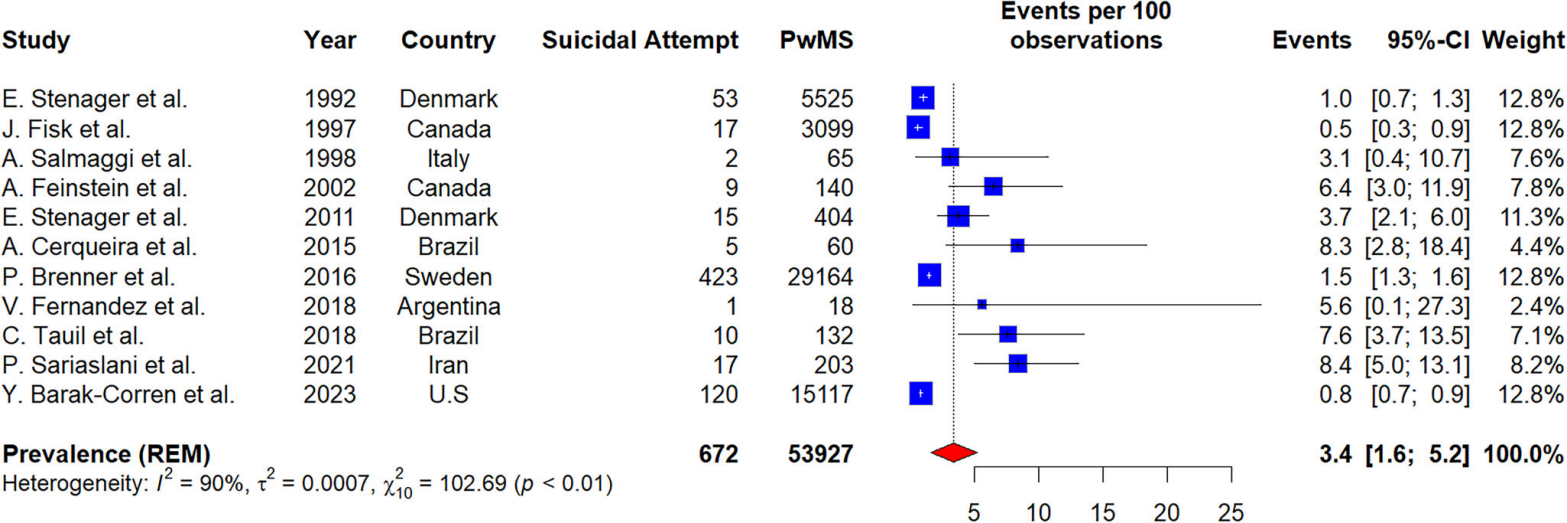
Prevalence of suicide attempt in PwMS.

The pooled prevalence of suicide attempts among PwMS was 3.4% (number of studies = 11, 95% CI: 1.6–5.2, I^2^ = 90%, *p*‐value for heterogeneity < 0.01) (Figure 3). Studies conducted in Europe reported a prevalence of 1.8% (95% CI: 0.6–3.0, I^2^ = 83%, *p*‐value for heterogeneity < 0.01). Studies from America reported a higher prevalence of 4.0% (95% CI: 0.8–7.1, I^2^ = 79%, *p*‐value for heterogeneity < 0.01). The single study from Asia (Iran) reported the highest prevalence of 8.4% (95% CI: 5.0–13.1). The differences between geographic subgroups were statistically significant (*p*‐value for subgroup difference < 0.01). Furthermore, meta‐regression indicated that age was not significantly associated with the prevalence of suicide attempts among PwMS.

#### 3.2.3 Suicide Mortality

**FIGURE 4:**
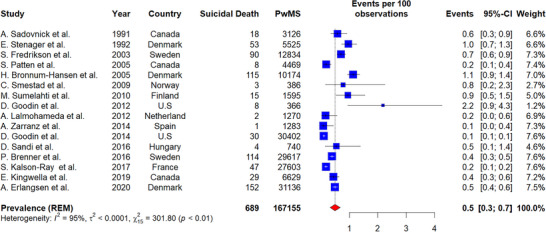
Prevalence of suicide mortality in PwMS.

The prevalence of suicide mortality among PwMS was 0.5% (number of studies = 16, 95% CI: 0.3–0.7, I^2^ = 95%, *p*‐value for heterogeneity < 0.01) (Figure 4). Studies conducted in America reported a prevalence of 0.4% (95% CI: 0.1–0.7, I^2^ = 89%, *p*‐value for heterogeneity < 0.01). Studies from Europe reported a prevalence of 0.5% (95% CI: 0.3–0.8, I^2^ = 95%, *p*‐value for heterogeneity < 0.01). The differences between geographic subgroups were not statistically significant (*p*‐value for subgroup difference = 0.37).

#### 3.2.4 Suicide Mortality Among Total Mortality

**FIGURE 5:**
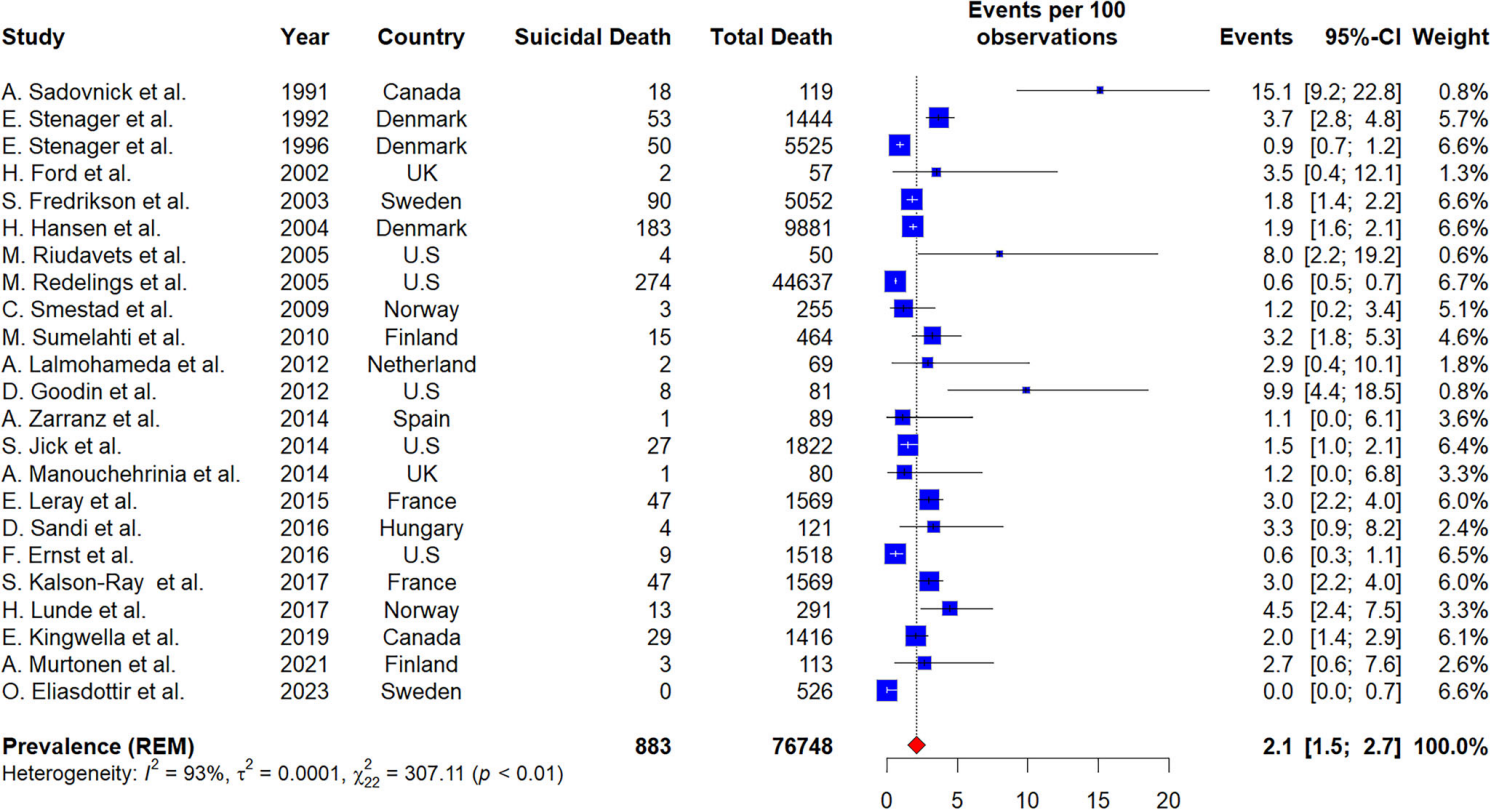
Prevalence of suicide mortality among total mortality in PwMS.

In PwMS, among total mortality, 2.1% was due to suicide (number of studies = 23, 95% CI: 1.5–2.7, I^2^ = 95%, *p*‐value for heterogeneity < 0.01) (Figure 5). This prevalence was higher in America (4.4%, 95% CI: 0.6–8.3, I^2^ = 89%, *p*‐value for heterogeneity < 0.01) than in Europe (2.1%, 95% CI: 1.5–2.8, I^2^ = 92%, *p*‐value for heterogeneity < 0.01) but the results were not significant (*p*‐value for subgroup difference = 0.24).

A summary of geographic variations in the prevalence of all suicide‐related outcomes is provided in Table 2. Other subgroup analyses are available as supplementary materials (Supplementary materials 2, Figures S1–S10).

**TABLE 2:** Prevalence of suicide‐related outcomes in PwMS across geographic regions.

Outcome	Europe	America	Asia	*p*‐value for subgroup difference
Suicide ideation	21.5 (8.2 − 34.8)	17.7 (11.6 − 23.9)	32.0 (21.9 − 42.1)	0.06
Suicide attempt	1.8 (0.6 − 3.0)	4.0 (0.8 − 7.1)	8.4 (5.0 − 13.1)	**< 0.01**
Suicide mortality	0.5 (0.3 − 0.8)	0.4 (0.1 − 0.7)	−	0.37
Suicide mortality among total mortality	2.1 (1.5 − 2.8)	4.4 (0.6 − 8.3)	−	0.24

### 3.3 Risk of Suicide Mortality in PwMS Compared to Healthy Controls

**FIGURE 6:**
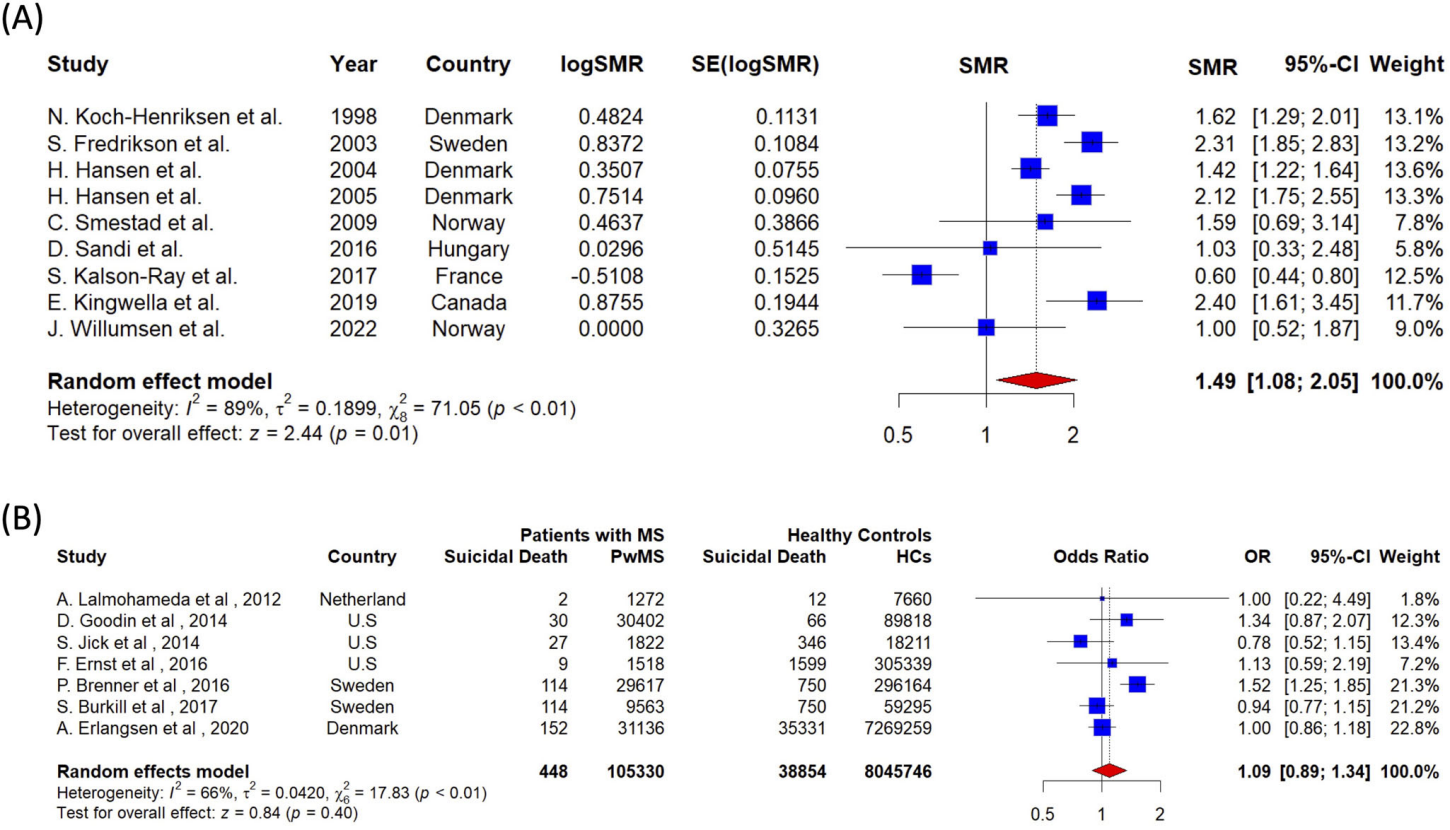
Risk of Suicide in PwMS: (A) SMR, (B) OR.

Due to diversity in effect size reporting among studies, different effect sizes was pooled separately. The pooled SMR for suicide mortality in PwMS was 1.49 (number of studies = 9, 95% CI: 1.08–2.05, I^2^ = 89%, *p*‐value for heterogeneity < 0.01), showing significantly higher suicide mortality in PwMS compared to healthy controls (Figure 6A). Conversely, the pooled OR for suicide mortality in PwMS compared to healthy controls showed insignificant results (number of studies = 7, OR = 1.09, 95% CI: 0.89–1.34, I^2^ = 66%, *p*‐value for heterogeneity < 0.01) (Figure 6B). RR and HR effect sizes were also pooled, and the results are available in supplementary materials (Supplementary materials 2, Figure S19,22).

To assess the potential for publication bias, we conducted both Egger's and Begg's tests and constructed funnel plots for each effect size. The results of these analyses indicated no significant evidence of publication bias across the included studies. The funnel plots exhibited symmetrical distributions, further supporting the absence of bias in the reported findings. Funnel plots are available in supplementary materials (Supplementary materials 2, Figure S11–15,17,20,23).

A sensitivity analysis was conducted to assess the heterogeneity among the included studies. In the analysis, no outliers were identified that significantly influenced the overall results (Supplementary materials 2, Figure S16,18,21,24).

## 4 Discussion

This systematic review and meta‐analysis highlights the substantial psychological burden faced by PwMS, as reflected by the notable prevalence of suicide ideation, attempts, and deaths. Among the outcomes analyzed, only suicide attempts showed statistically significant regional differences, with higher prevalence observed in America compared to Europe. In contrast, regional variations in suicide ideation, suicide mortality, and the proportion of suicide‐related deaths were not statistically significant. Compared to the general population, PwMS showed a significantly higher risk of suicide mortality. These findings emphasize the need for targeted mental health strategies and integration of psychological care into routine MS management.

Given the high prevalence of suicidality in PwMS, it is important to contextualize our findings by comparing them with prior studies. In our study, approximately one‐fifth of PwMS reported experiencing suicide ideation. Therefore, at first it is essential to consider potential risk factors for these thoughts. Viner et al. found that older age, low self‑efficacy, and MS‑related physical symptoms (like bowel/bladder dysfunction and speech difficulties) were among the strongest predictors of suicide ideation in PwMS, even after accounting for depressive symptoms (Viner et al. 2014). Furthermore, another cross‐sectional study in 2020 demonstrated that suicide ideation was significantly more likely among individuals reporting higher anxiety and depression scores or experiencing anticipated stigma from friends and family (Asgarian et al. 2020). Therefore, assessing and considering these risk factors may assist clinicians in better supporting and managing their patients.

It is well established that suicide ideation could precede suicide attempts. The elevated prevalence of suicide attempts observed in our analysis is consistent with the findings of a 2023 meta‐analysis, which reported that 4.1% of PwMS had attempted suicide (d'Andrade et al. 2023). This aligns with studies emphasizing the psychological burden of MS, as it is well known that suicide attempts are associated with psychological states (Williams 1997, Joiner 2005, Klonsky and May 2015). In a systematic review and meta‐analysis by Boeschoten et al. (2017), which included a total of 87,756 PwMS, the pooled prevalence of depression was roughly one‑third of PwMS, while anxiety was reported in nearly one‑quarter of cases. Furthermore, their subgroup analysis also showed a comparatively lower frequency of depressive disorders in studies conducted in Europe (Boeschoten et al. 2017), which may partially explain our finding of significantly lower suicide attempt prevalence in this region.

Geographical subgroup analysis was performed to explore potential regional differences in suicide ideation prevalence that may be driven by variations in healthcare systems, cultural attitudes towards mental health, and reporting practices. However, unlike what was observed for suicide attempts, we did not find a significant geographic difference in suicide ideation prevalence, despite the well‐established association between depression and suicide ideation. This apparent inconsistency may be influenced by sociocultural and systemic differences across regions. For example, individuals in some countries may be more willing to disclose suicidal thoughts in anonymous surveys but less likely to act on them or report suicide attempts due to social stigma, fear of hospitalization, or limited access to emergency psychiatric care. Conversely, higher reporting of suicide attempts in America may reflect broader access to mental health services, differing thresholds for classification, or reduced stigma surrounding disclosure of psychiatric crises. These contextual factors may influence both actual behavior and its documentation across studies.

Notably, the highest suicide attempt prevalence in our analysis was reported in the only included Asian study, which was conducted in Iran and found a prevalence of 8.4%. This prevalence is substantially higher than the pooled estimates for America (4.0%) and Europe (1.8%), suggesting that suicide risk in PwMS may be significantly elevated in certain non‐Western contexts. However, this finding should be interpreted with caution, as it may be influenced by culturally specific factors, differences in healthcare infrastructure, or methodological variation. Qualitative elements such as mental health stigma, religious or legal consequences of self‐harm, and divergent diagnostic or reporting practices may all contribute to elevated prevalence figures. Although a single data point does not allow for broader generalizations across the Asian continent, it highlights the need for more regionally representative data and culturally contextualized research on suicide risk in PwMS.

Lastly, variability in suicide assessment tools across the studies reporting suicide attempts may have contributed to inconsistencies in prevalence estimates. Instruments such as the BSSI (Beck and Steer 1993) and International Classification of Diseases‐9 differ in how they define, detect, and classify suicide attempts, leading to potential underreporting or misclassification. Importantly, no instrument has been psychometrically validated specifically for detecting suicide attempts in PwMS. Most studies rely on tools developed for the general psychiatric population, which may not fully capture the unique psychological and neurological context of PwMS. This methodological heterogeneity, as summarized in Table 1, likely explains some of the variability in suicide attempt prevalence and underscores the need for standardized, MS‐appropriate assessment tools in future research. In line with this, our analysis also revealed that the prevalence of suicide ideation varied significantly depending on the type of instrument used. Particularly, instruments specifically designed to measure suicide ideation produced higher estimates compared to general screening tools. This highlights the critical impact of measurement sensitivity on reported suicide ideation prevalence and suggests that future research should prioritize the use of validated, suicidality‐focused instruments for accurate detection and monitoring.

The higher suicide mortality risk among PwMS (SMR = 1.49) is in line with results from large cohort studies carried out in Denmark, which found SMRs of 1.83 (Stenager et al. 1992), 1.62 (Koch‐Henriksen et al. 1998), and 2.12 (Brønnum‐Hansen et al. 2005), respectively. Similar patterns were seen in Sweden (SMR = 2.3) (Fredrikson et al. 2003) and Finland (SMR = 1.7) (Sumelahti et al. 2010), where the risk of suicide was almost twice as high for PwMS as for the general population. Additionally, as previously mentioned, the most recent meta‐analysis found that PwMS have a 72% higher risk of suicide than the general population, with a pooled SRR of 1.72 (Shen et al. 2019). This result is in line with a 2016 meta‐analysis by Manouchehrinia et al., which revealed that PwMS have a risk of suicide mortality that is more than twice that of the general population, with a pooled SMR of 2.13 (Manouchehrinia et al. 2016). Both meta‐analyses support our findings. The SMR of our study was lower than that of the 2016 meta‐analysis (Manouchehrinia et al. 2016) for several reasons (SMR = 1.49 vs. 2.13). For instance, improvements in mental health awareness, early detection, and interventions targeting psychiatric comorbidities in PwMS over the past decade may have reduced the risk of suicide mortality. Advances in MS treatment and multidisciplinary care approaches may have reduced psychological distress and suicide ideation, thereby facilitating improved disease management. Additionally, regional differences in healthcare access and support networks among the recent studies might have had an impact on the pooled SMR in our analysis.

While our pooled SMR (1.49) indicates a significantly elevated risk of suicide mortality among PwMS, the pooled OR (1.09) was not statistically significant. This discrepancy can be explained by several methodological differences. First, case‐control studies reporting ORs frequently lacked adjustment for critical confounders, such as depression, psychiatric comorbidities, and socioeconomic status (e.g., income, education, employment), which may potentially be associated with suicide risk. When unadjusted, these factors may bias effect estimates toward the null. Second, SMRs are typically derived from large‐scale cohort studies with extended follow‐up, which allow for the detection of long‐term suicide risk and adjustment for time‐varying confounders. In contrast, ORs are often based on short‐term, cross‐sectional data that do not account for the cumulative burden of MS. In our analysis, SMR studies generally had larger sample sizes, longer follow‐up periods, and more thorough confounder control, factors that likely contribute to the robustness of SMR results compared to ORs (Supplementary materials 3, Table S2). Suicide remains a significant but under recognized cause of death in PwMS. A 2020 cohort study found that among various causes of death, suicide had one of the highest SMRs (SMR = 2.40), underscoring its critical contribution to overall mortality in this population (Kingwell et al. 2020).

Our findings highlight an urgent need for proactive suicide prevention strategies in PwMS, given the substantial burden of suicidality in this population. Multidisciplinary collaboration among neurologists, psychiatrists, and primary care providers is essential to facilitate routine suicide risk assessments and timely interventions, including evidence‐based psychotherapies and pharmacologic treatments. Although no screening tool has been psychometrically validated specifically for assessing suicide risk in PwMS, several instruments developed for the general psychiatric population are commonly used in MS research and clinical settings. These include the BSSI (Beck and Steer 1993) and item 9 of the Patient Health Questionnaire‐9 (Kroenke et al. 2001). Integrating such tools into standard MS care, in conjunction with clinical judgment and individualized psychiatric evaluation, may enhance early detection and intervention. Furthermore, cognitive behavioral therapy (CBT) has demonstrated efficacy in reducing depression and suicide ideation and should be considered as a frontline intervention (Zhao and Wang 2025). Public health campaigns to reduce mental health stigma, particularly in regions with elevated suicide risk, may further promote help‐seeking behavior and improve access to care.

One of the main strengths of this study is its comprehensive and methodologically rigorous approach. By synthesizing data from 64 studies involving over 200,000 individuals with MS across 19 countries, we were able to generate robust pooled estimates for suicide ideation, suicide attempts, and suicide mortality. However, several limitations should be acknowledged. First, high heterogeneity was observed across the pooled estimates in this study. Although we conducted multiple subgroup and meta‐regression analyses (e.g., based on age, disease duration, and assessment tools), the sources of heterogeneity could not be fully identified. Notably, high statistical heterogeneity is an inherent characteristic of prevalence meta‐analyses, often resulting from variations in study populations, methodologies, and settings (Migliavaca et al. 2022). Moreover, meta‐regression analyses were limited to variables with sufficient data, such as age and disease duration, while other potentially relevant factors could not be assessed. Another methodological limitation of this study was the post‐hoc nature of subgroup analyses, particularly those based on thresholds. These categorizations were not pre‐specified in the PROSPERO protocol and were determined during the analysis phase based on available data distributions and precedent in the MS literature. While such exploratory analyses can offer useful insights, post‐hoc decisions inherently carry a risk of data‐driven bias and may increase the likelihood of spurious associations. Therefore, the findings from these subgroup comparisons should be interpreted with caution and considered hypothesis‐generating rather than confirmatory. Furthermore, there is a clear geographic imbalance in the data: most studies originated from Europe (n = 30) and North America (n = 25), whereas only a limited number came from Asia (n = 5) and South America (n = 4), with no representation from Africa or Southeast Asia. This underrepresentation limits the global applicability of our findings and hampers understanding of how cultural, economic, and healthcare system differences may influence suicide risk in PwMS. Future research should prioritize generating high‐quality data from these underserved regions using culturally adapted, standardized assessment tools.

Another important limitation is the lack of conclusive evidence regarding the role of disease‐modifying therapies (DMTs) in suicide risk among PwMS. Although β‐interferon, one of the most widely used DMTs, has been implicated as a potential trigger for suicidal behavior in rare instances (Nagesh et al. 2015, Mumoli et al. 2013), current evidence remains limited and inconclusive. For example, a case report by Mumoli et al. described a suicide attempt during natalizumab treatment, suggesting a possible, though extremely rare, and association with suicide ideation (Mumoli et al. 2013). However, isolated case reports cannot establish causality or generalize risk. In contrast, a 2018 systematic review concluded that DMTs are not associated with an increased risk of adverse psychiatric effects such as suicide attempt, suicide ideation, suicidal behavior, and suicide in PwMS (Gasim et al. 2018). Overall, no broad or definitive evidence currently links DMTs directly to increased suicide risk beyond the influence of underlying disease burden and psychiatric comorbidities. Given the widespread and long‐term use of DMTs in PwMS and the psychological vulnerability of this population, further longitudinal studies are needed to better understand any potential neuropsychiatric effects of these therapies.

In summary, our review provides strong evidence that PwMS face a higher risk of suicide compared to the general population, emphasizing the need for integrating mental health services into routine MS care. Despite advancements in MS treatment, mental health care for PwMS remains inadequate, as evidenced by persistently elevated SMR. Future studies should explore the impact of DMTs on suicide risk, as well as investigate potential protective factors such as social support, resilience, and CBT‐based interventions. Additionally, the development of standardized suicide risk assessment tools specific to PwMS is necessary to improve comparability across studies and facilitate early identification of at‐risk individuals. Future interventions should not only focus on mental health support but also explore the role of social support, lifestyle factors, and treatment adherence in reducing suicide risk.

## Author Contributions

**Omid Mirmosayyeb**: conceptualization, methodology, supervision, writing – review and editing, project administration, validation. **Homa Khodadadi**: data curation, writing – review and editing. **Aynaz Mohammadi**: writing – review and editing, writing – original draft. **Motahareh Abbasi**: writing – review and editing, supervision. **Saeed Vaheb**: writing – review and editing, data curation. **Mohammad Yazdan Panah**: writing – original draft, writing – review and editing, data curation, formal analysis, visualization, methodology, software. **Mohammad Mohammadi**: writing – review and editing, writing – original draft, project administration, conceptualization, supervision, methodology. **Vahid Shaygannejad**: supervision, writing – review and editing, conceptualization, methodology, validation.

## Ethics Statement

The authors have nothing to report.

## Peer Review

The peer review history for this article is available at https://publons.com/publon/10.1002/brb3.70839.

## Supporting information



Supplementary materials 1. Search strategy for each database

Supplementary materials 2. Subgroup analyses, sensitivity analyses, and funnel plots

Supplementary materials 3. Comparison of Study Characteristics between OR and SMR Studies

## Data Availability

This is a systematic review and meta‐analysis article and all relevant data are within the manuscript and tables.
