# Supplementary material for: Suicide Ideation, Attempts, and Mortality in Multiple Sclerosis: A Systematic Review and Meta‐Analysis
Source: Brain Behav. 2025 Sep 9;15(9):e70839. doi: 10.1002/brb3.70839 (PMC12417961; doi:10.1002/brb3.70839)
Supplement: Supplementary file 1 — Supplementary materials 1. Search strategy for each database [file BRB3-15-e70839-s001.docx]

Table 1. Search strategy for each database

| Syntax | all | tiab |
| --- | --- | --- |
| Pubmed/MEDLINE | | |
| ("Multiple sclerosis"[All Fields] OR "Sclerosis, Multiple"[All Fields] OR "Sclerosis, Disseminated"[All Fields] OR "Disseminated Sclerosis"[All Fields] OR "MS (Multiple Sclerosis)"[All Fields]) AND ("Suicide"[All Fields] OR "Suicidal Ideation"[All Fields] OR "Suicide Prevention"[All Fields] OR "Suicide, Assisted"[All Fields] OR "Suicide, Attempted"[All Fields] OR "Suicide, Completed"[All Fields]) | 246 |  |
|  |  |  |
| Scopus | | |
| (ALL("Multiple sclerosis") OR ALL("Sclerosis, Multiple") OR ALL("Sclerosis, Disseminated") OR ALL("Disseminated Sclerosis") OR ALL("MS (Multiple Sclerosis)")) AND (ALL("Suicide") OR ALL("Suicidal Ideation") OR ALL("Suicide Prevention") OR ALL("Suicide, Assisted") OR ALL("Suicide, Attempted") OR ALL("Suicide, Completed")) |  |  |
| (TITLE-ABS-KEY("Multiple sclerosis") OR TITLE-ABS-KEY("Sclerosis, Multiple") OR TITLE-ABS-KEY("Sclerosis, Disseminated") OR TITLE-ABS-KEY("Disseminated Sclerosis") OR TITLE-ABS-KEY("MS (Multiple Sclerosis)")) AND (TITLE-ABS-KEY("Suicide") OR TITLE-ABS-KEY("Suicidal Ideation") OR TITLE-ABS-KEY("Suicide Prevention") OR TITLE-ABS-KEY("Suicide, Assisted") OR TITLE-ABS-KEY("Suicide, Attempted") OR TITLE-ABS-KEY("Suicide, Completed")) | 647 |  |
| Web of Science | | |
| TS=("Multiple sclerosis" OR "Sclerosis, Multiple" OR "Sclerosis, Disseminated" OR "Disseminated Sclerosis" OR "MS (Multiple Sclerosis)") AND TS=("Suicide" OR "Suicidal Ideation" OR "Suicide Prevention" OR "Suicide, Assisted" OR "Suicide, Attempted" OR "Suicide, Completed") | 707 |  |
| ALL=("Multiple sclerosis" OR "Sclerosis, Multiple" OR "Sclerosis, Disseminated" OR "Disseminated Sclerosis" OR "MS (Multiple Sclerosis)") AND ALL=("Suicide" OR "Suicidal Ideation" OR "Suicide Prevention" OR "Suicide, Assisted" OR "Suicide, Attempted" OR "Suicide, Completed") |  | 196 |
| Embase | | |
| ('multiple sclerosis' OR 'sclerosis, multiple' OR 'sclerosis, disseminated' OR 'disseminated sclerosis' OR 'ms (multiple sclerosis)')  AND  ('suicide' OR 'suicidal ideation' OR 'suicide prevention' OR 'suicide, assisted' OR 'suicide, attempted' OR 'suicide, completed') | 892 |  |
| ('multiple sclerosis':ti,ab OR 'sclerosis, multiple':ti,ab OR 'sclerosis, disseminated':ti,ab OR 'disseminated sclerosis':ti,ab OR 'ms (multiple sclerosis)':ti,ab)  AND  ('suicide':ti,ab OR 'suicidal ideation':ti,ab OR 'suicide prevention':ti,ab OR 'suicide, assisted':ti,ab OR 'suicide, attempted':ti,ab OR 'suicide, completed':ti,ab) |  |  |
| Google scholar | | |
| ("Multiple sclerosis" OR "Sclerosis, Multiple" OR "Sclerosis, Disseminated" OR "Disseminated Sclerosis" OR "MS (Multiple Sclerosis)") AND ("Tremor" OR "Intention Tremor" OR "Intention Tremors" OR "Tremor, Intention" OR "Darkness Tremor" OR "Darkness Tremors" OR "Tremor, Darkness" OR "Pill Rolling Tremor" OR "Pill Rolling Tremors" OR "Tremor, Pill Rolling" OR "Fine Tremor" OR "Fine Tremors" OR "Tremor, Fine" OR "Intermittent Tremor" OR "Intermittent Tremors" OR "Tremor, Intermittent" OR "Involuntary Quiver" OR "Involuntary Quivers" OR "Quiver, Involuntary" OR "Massive Tremor" OR "Massive Tremors" OR "Tremor, Massive" OR "Passive Tremor" OR "Passive Tremors" OR "Tremor, Passive" OR "Persistent Tremor" OR "Persistent Tremors" OR "Tremor, Persistent" OR "Resting Tremor" OR "Resting Tremors" OR "Tremor, Resting" OR "Rest Tremor" OR "Rest Tremors" OR "Tremor, Rest" OR "Tremor, Perioral" OR "Perioral Tremor" OR "Perioral Tremors" OR "Tremor, Semirhythmic" OR "Semirhythmic Tremor" OR "Semirhythmic Tremors" OR "Saturnine Tremor" OR "Saturnine Tremors" OR "Tremor, Saturnine" OR "Senile Tremor" OR "Senile Tremors" OR "Tremor, Senile" OR "Static Tremor" OR "Static Tremors" OR "Tremor, Static" OR "Tremor, Limb" OR "Limb Tremor" OR "Limb Tremors" OR "Tremor, Muscle" OR "Muscle Tremor" OR "Muscle Tremors" OR "Tremor, Neonatal" OR "Neonatal Tremor" OR "Neonatal Tremors" OR "Tremor, Nerve" OR "Nerve Tremor" OR "Nerve Tremors" OR "Action Tremor" OR "Action Tremors" OR "Tremor, Action" OR "Coarse Tremor" OR "Coarse Tremors" OR "Tremor, Coarse" OR "Continuous Tremor" OR "Continuous Tremor" OR "Tremor, Continuous") | 24200 |  |
|  |  |  |

("Multiple sclerosis"OR "Sclerosis, Multiple" OR "Sclerosis, Disseminated" OR "Disseminated Sclerosis" OR "MS (Multiple Sclerosis)") AND (Dysgeusias OR Taste, Distorted OR Distorted Taste OR Taste, Altered OR Altered Taste OR Parageusia OR Parageusias OR Taste Disorders OR Dysgeusia OR Ageusia OR Taste Disorder OR Taste Disorder, Primary OR Primary Taste Disorder OR Primary Taste Disorders OR Taste Disorders, Primary OR Taste Disorder, Secondary OR Secondary Taste Disorder OR Secondary Taste Disorders OR Taste Disorders, Secondary OR Taste, Metallic OR Metallic Taste OR Metallic Tastes OR Tastes, Metallic OR Taste Disorder, Anterior Tongue OR Taste Disorder, Posterior Tongue OR Taste Disorder, Primary, Bitter OR Taste Disorder, Primary, Salt OR Taste Disorder, Primary, Sweet OR Taste Disorder, Secondary, Bitter OR Taste Disorder, Secondary, Salt OR Taste Disorder, Secondary, Sweet OR Taste Dysfunction OR Dysfunction, Taste OR Loss of Taste OR Taste Loss OR Ageusia, Hysterical OR Ageusias, Hysterical OR Hysterical Ageusia OR Hysterical Ageusias OR Hypogeusia OR Hypogeusias OR Taste-Blindness OR Taste Blindness OR Gustatory OR Perception, Taste OR Taste Perceptions OR Gustatory Perception OR Perception, Gustatory OR Gustatory Response OR Gustatory Responses OR Response, Gustatory OR Gustatory)
