# Supplementary material for: Suicide Ideation, Attempts, and Mortality in Multiple Sclerosis: A Systematic Review and Meta‐Analysis
Source: Brain Behav. 2025 Sep 9;15(9):e70839. doi: 10.1002/brb3.70839 (PMC12417961; doi:10.1002/brb3.70839)

Figure S1. Prevalence of suicide ideation in PwMS: Sample size subgroup

Study	Year	Country	Suicidal Ideation	PwMS	Events	95%-CI	Weight
**Sample Size = Less than 500**							
V. Eklund et al.	1991	US	27	125	21.6	[14.7; 29.8]	5.8%
A. Feinstein et al.	1999	Canada	33	152	21.7	[15.4; 29.1]	5.9%
S. Quesnel et al.	2004	Canada	40	140	28.6	[21.3; 36.8]	5.8%
S. Askey-Jones et al.	2012	UK	30	90	33.3	[23.7; 44.1]	5.4%
V. Lewis et al.	2016	UK	26	75	34.7	[24.0; 46.5]	5.2%
Y. Alamri et al.	2016	Saudi Arabia	3	24	12.5	[2.7; 32.4]	4.8%
K. Altura et al.	2016	Canada	12	148	8.1	[4.3; 13.7]	6.2%
S. Ariapooran et al.	2016	Iran	24	79	30.4	[20.5; 41.8]	5.3%
A. Romaniuc et al.	2020	Romania	35	349	10.0	[7.1; 13.7]	6.3%
F. Asgarian et al.	2020	Iran	100	276	36.2	[30.6; 42.2]	6.0%
V. Vacaras et al.	2020	Romania	16	146	11.0	[6.4; 17.2]	6.1%
P. Sariaslani et al.	2021	Iran	61	203	30.0	[23.8; 36.9]	6.0%
S. Pouradeli et al.	2021	Iran	94	202	46.5	[39.5; 53.7]	5.9%
**Prevalence (REM)**			501	2009	24.8	[18.2; 31.4]	74.7%
Heterogeneity: $I^2 = 94\%$, $\tau^2 = 0.0133$, $\chi^2_{12} = 202.13$ ($p < 0.01$)							
**Sample Size = More than 500**							
C. Berkman et al.	1999	US	154	505	30.5	[26.5; 34.7]	6.2%
R. Viner et al.	2014	Canada	56	630	8.9	[6.8; 11.4]	6.4%
L. Dickstein et al.	2014	US	562	3823	14.7	[13.6; 15.9]	6.4%
L. Knowles et al.	2020	US	59	573	10.3	[7.9; 13.1]	6.3%
**Prevalence (REM)**			831	5531	16.0	[6.4; 25.6]	25.3%
Heterogeneity: $I^2 = 97\%$, $\tau^2 = 0.0093$, $\chi^2_3 = 95.14$ ($p < 0.01$)							
**Prevalence (REM)**			**1332**	**7540**	**22.6**	**[16.9; 28.3]**	**100.0%**
Heterogeneity: $I^2 = 95\%$, $\tau^2 = 0.0131$, $\chi^2_{16} = 332.95$ ($p < 0.01$)							
Test for subgroup differences: $\chi^2_1 = 2.21$, df = 1 ($p = 0.14$)							

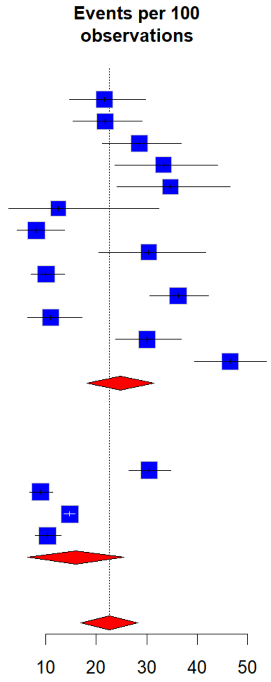

Figure S2. Prevalence of suicide ideation in PwMS: Continent subgroup

Study	Year	Country	Suicidal Ideation	PwMS	Events	95%-CI	Weight
**Continent = America**							
V. Eklund et al.	1991	US	27	125	21.6	[14.7; 29.8]	5.8%
A. Feinstein et al.	1999	Canada	33	152	21.7	[15.4; 29.1]	5.9%
C. Berkman et al.	1999	US	154	505	30.5	[26.5; 34.7]	6.2%
S. Quesnel et al.	2004	Canada	40	140	28.6	[21.3; 36.8]	5.8%
R. Viner et al.	2014	Canada	56	630	8.9	[6.8; 11.4]	6.4%
L. Dickstein et al.	2014	US	562	3823	14.7	[13.6; 15.9]	6.4%
K. Altura et al.	2016	Canada	12	148	8.1	[4.3; 13.7]	6.2%
L. Knowles et al.	2020	US	59	573	10.3	[7.9; 13.1]	6.3%
**Prevalence (REM)**			943	6096	17.7	[11.6; 23.9]	49.0%
Heterogeneity: $I^2 = 94\%$, $\tau^2 = 0.0073$, $\chi^2_7 = 126.04$ ($p < 0.01$)							
**Continent = Europe**							
S. Askey-Jones et al.	2012	UK	30	90	33.3	[23.7; 44.1]	5.4%
V. Lewis et al.	2016	UK	26	75	34.7	[24.0; 46.5]	5.2%
A. Romaniuc et al.	2020	Romania	35	349	10.0	[7.1; 13.7]	6.3%
V. Vacaras et al.	2020	Romania	16	146	11.0	[6.4; 17.2]	6.1%
**Prevalence (REM)**			107	660	21.5	[8.2; 34.8]	23.0%
Heterogeneity: $I^2 = 92\%$, $\tau^2 = 0.0168$, $\chi^2_3 = 36.32$ ($p < 0.01$)							
**Continent = Asia**							
Y. Alamri et al.	2016	Saudi Arabia	3	24	12.5	[2.7; 32.4]	4.8%
S. Ariapooran et al.	2016	Iran	24	79	30.4	[20.5; 41.8]	5.3%
F. Asgarian et al.	2020	Iran	100	276	36.2	[30.6; 42.2]	6.0%
P. Sariaslani et al.	2021	Iran	61	203	30.0	[23.8; 36.9]	6.0%
S. Pouradeli et al.	2021	Iran	94	202	46.5	[39.5; 53.7]	5.9%
**Prevalence (REM)**			282	784	32.0	[21.9; 42.1]	27.9%
Heterogeneity: $I^2 = 84\%$, $\tau^2 = 0.0113$, $\chi^2_4 = 25.25$ ($p < 0.01$)							
**Prevalence (REM)**			**1332**	**7540**	**22.6**	**[16.9; 28.3]**	**100.0%**
Heterogeneity: $I^2 = 95\%$, $\tau^2 = 0.0131$, $\chi^2_{16} = 332.95$ ($p < 0.01$)							
Test for subgroup differences: $\chi^2_2 = 5.64$, df = 2 ($p = 0.06$)							

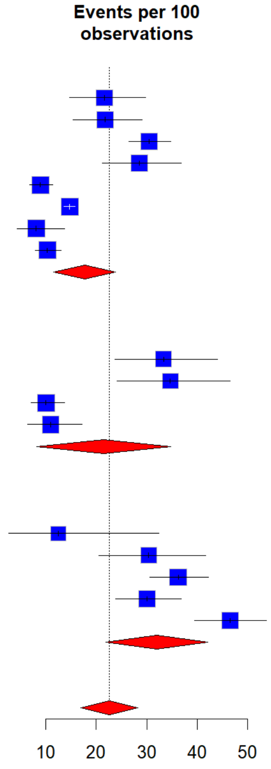

Figure S3. Prevalence of suicide ideation in PwMS: Age subgroup

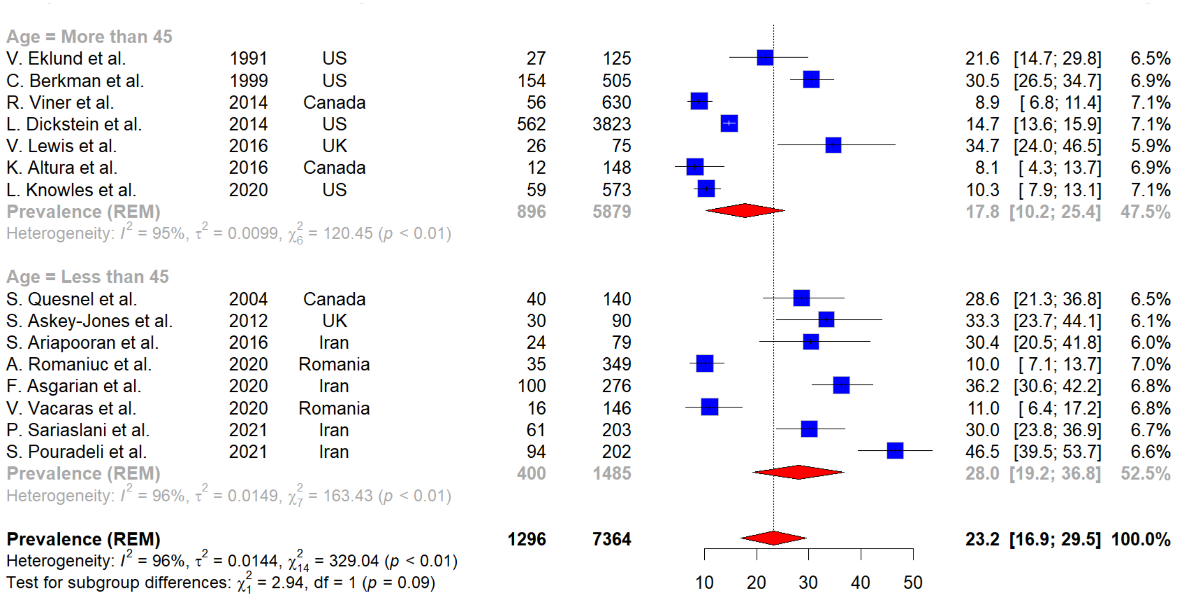

Figure S4. Prevalence of suicide ideation in PwMS: Suicide ideation assessment tool subgroup

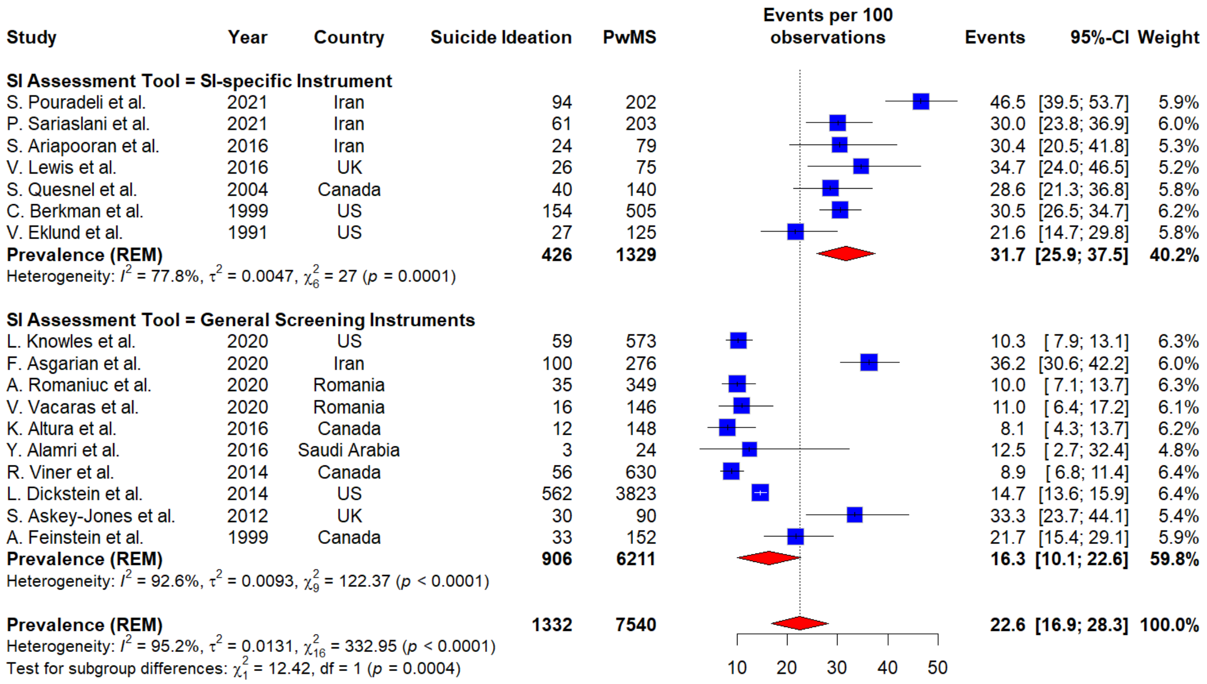

SI: Suicide ideation

Figure S5. Prevalence of suicide attempt in PwMS: Sample size subgroup

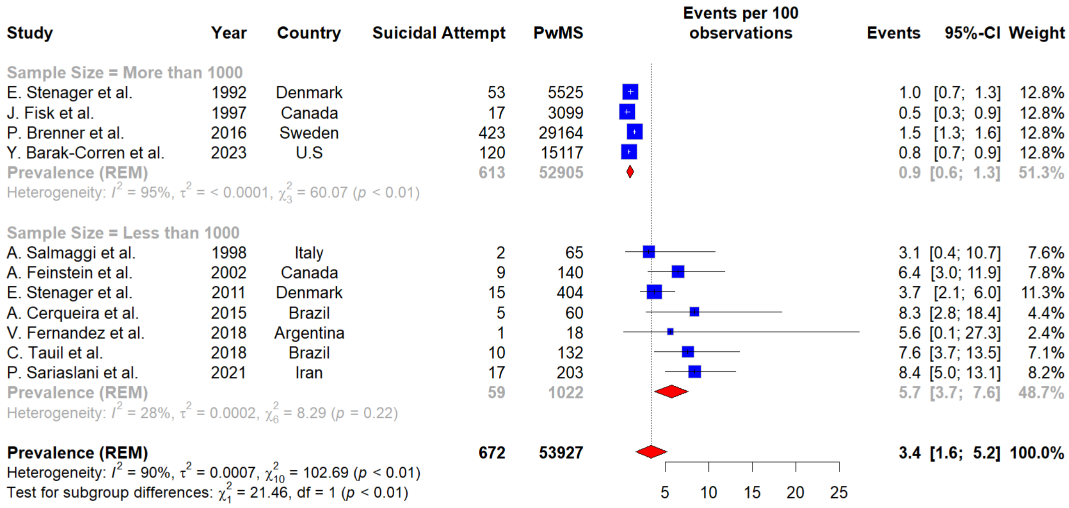

Figure S6. Prevalence of suicide attempt in PwMS: Continent subgroup

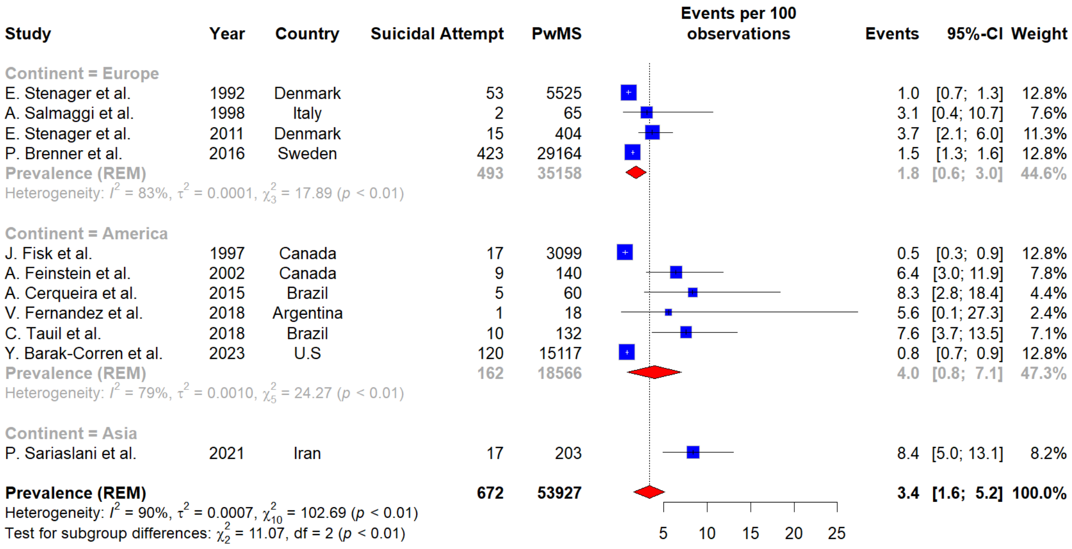

Figure S7. Prevalence of suicide mortality in PwMS: Sample size subgroup

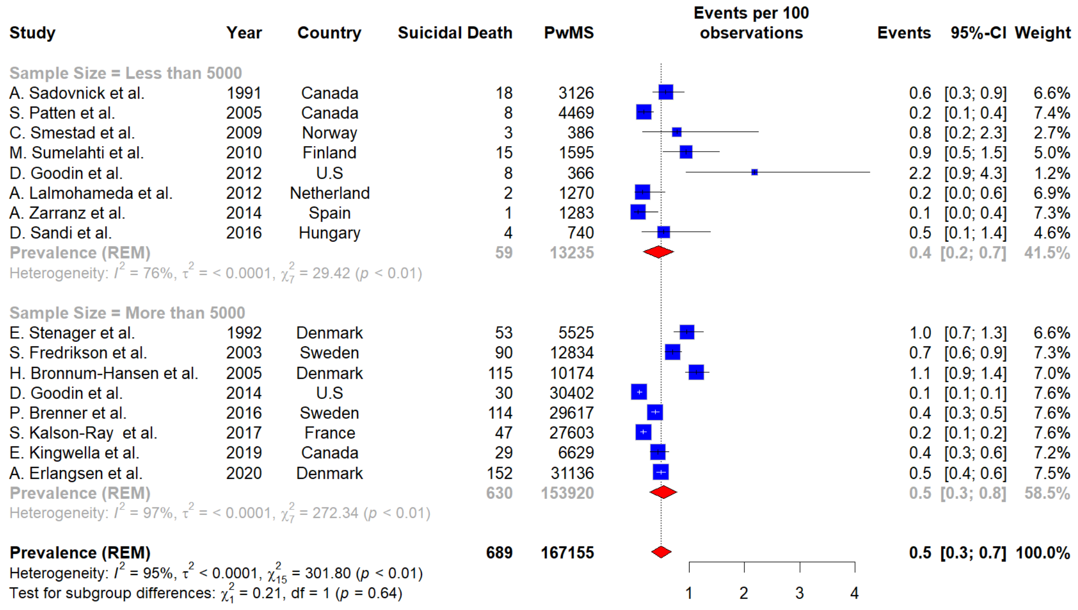

Figure S8. Prevalence of suicide mortality in PwMS: Continent subgroup

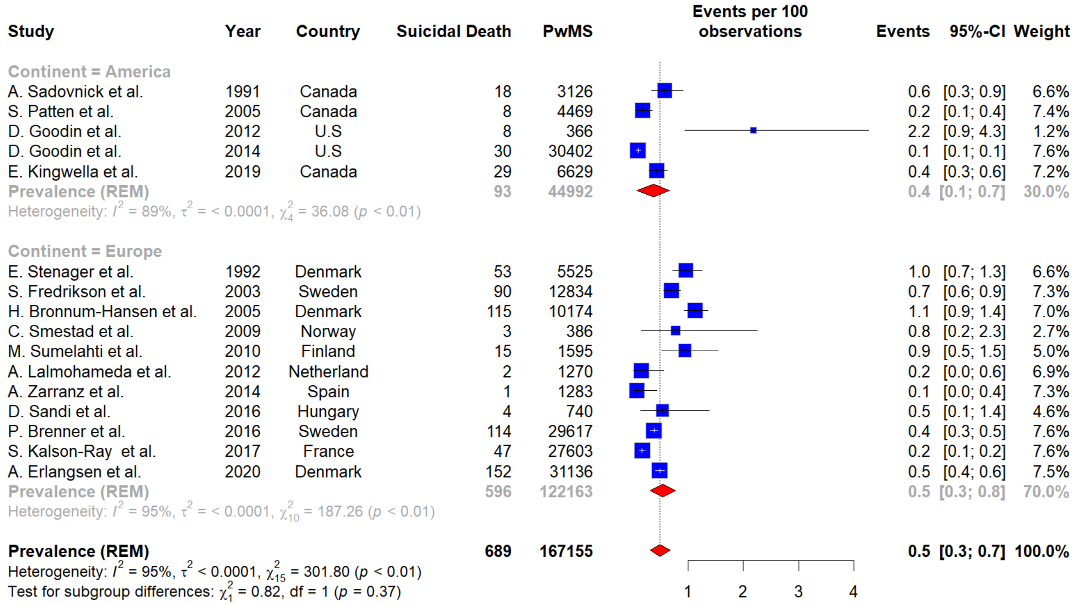

Figure S9. Prevalence of suicide mortality among total mortality in PwMS: Sample size subgroup

Study	Year	Country	Suicidal Death	Total Death	Events	95%-CI	Weight
**Sample Size = Less than 1000**							
A. Sadovnick et al.	1991	Canada	18	119	15.1	[9.2; 22.8]	0.8%
H. Ford et al.	2002	UK	2	57	3.5	[0.4; 12.1]	1.3%
M. Riudavets et al.	2005	U.S	4	50	8.0	[2.2; 19.2]	0.6%
C. Smestad et al.	2009	Norway	3	255	1.2	[0.2; 3.4]	5.1%
M. Sumelahti et al.	2010	Finland	15	464	3.2	[1.8; 5.3]	4.6%
A. Lalmohameda et al.	2012	Netherland	2	69	2.9	[0.4; 10.1]	1.8%
D. Goodin et al.	2012	U.S	8	81	9.9	[4.4; 18.5]	0.8%
A. Manouchehrinia et al.	2014	UK	1	80	1.2	[0.0; 6.8]	3.3%
A. Zarranz et al.	2014	Spain	1	89	1.1	[0.0; 6.1]	3.6%
D. Sandi et al.	2016	Hungary	4	121	3.3	[0.9; 8.2]	2.4%
H. Lunde et al.	2017	Norway	13	291	4.5	[2.4; 7.5]	3.3%
A. Murtonen et al.	2021	Finland	3	113	2.7	[0.6; 7.6]	2.6%
O. Eliasdottir et al.	2023	Sweden	0	526	0.0	[0.0; 0.7]	6.6%
**Prevalence (REM)**			**74**	**2315**	**3.2**	**[1.6; 4.8]**	**36.8%**
Heterogeneity: $I^2 = 84\%$, $\tau^2 = 0.0006$, $\chi^2_{12} = 74.4$ ($p < 0.01$)							
**Sample Size = More than 1000**							
E. Stenager et al.	1992	Denmark	53	1444	3.7	[2.8; 4.8]	5.7%
E. Stenager et al.	1996	Denmark	50	5525	0.9	[0.7; 1.2]	6.6%
S. Fredrikson et al.	2003	Sweden	90	5052	1.8	[1.4; 2.2]	6.6%
H. Hansen et al.	2004	Denmark	183	9881	1.9	[1.6; 2.1]	6.6%
M. Redelings et al.	2005	U.S	274	44637	0.6	[0.5; 0.7]	6.7%
S. Jick et al.	2014	U.S	27	1822	1.5	[1.0; 2.1]	6.4%
E. Leray et al.	2015	France	47	1569	3.0	[2.2; 4.0]	6.0%
F. Ernst et al.	2016	U.S	9	1518	0.6	[0.3; 1.1]	6.5%
S. Kalson-Ray et al.	2017	France	47	1569	3.0	[2.2; 4.0]	6.0%
E. Kingwella et al.	2019	Canada	29	1416	2.0	[1.4; 2.9]	6.1%
**Prevalence (REM)**			**809**	**74433**	**1.8**	**[1.2; 2.5]**	**63.2%**
Heterogeneity: $I^2 = 96\%$, $\tau^2 = < 0.0001$, $\chi^2_9 = 217.61$ ($p < 0.01$)							
**Prevalence (REM)**			**883**	**76748**	**2.1**	**[1.5; 2.7]**	**100.0%**
Heterogeneity: $I^2 = 93\%$, $\tau^2 = 0.0001$, $\chi^2_{22} = 307.11$ ($p < 0.01$)							
Test for subgroup differences: $\chi^2_1 = 2.52$, df = 1 ($p = 0.11$)							

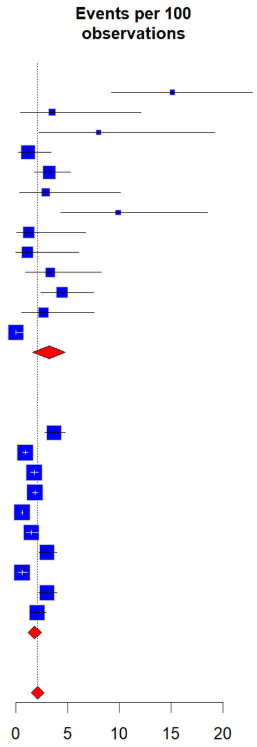

Figure S10. Prevalence of suicide mortality among total mortality in PwMS: Continent subgroup

Study	Year	Country	Suicidal Death	Total Death	Events	95%-CI	Weight
**Continent = America**							
A. Sadovnick et al.	1991	Canada	18	119	15.1	[9.2; 22.8]	0.8%
M. Riudavets et al.	2005	U.S	4	50	8.0	[2.2; 19.2]	0.6%
M. Redelings et al.	2005	U.S	274	44637	0.6	[0.5; 0.7]	6.7%
D. Goodin et al.	2012	U.S	8	81	9.9	[4.4; 18.5]	0.8%
S. Jick et al.	2014	U.S	27	1822	1.5	[1.0; 2.1]	6.4%
F. Ernst et al.	2016	U.S	9	1518	0.6	[0.3; 1.1]	6.5%
E. Kingwella et al.	2019	Canada	29	1416	2.0	[1.4; 2.9]	6.1%
**Prevalence (REM)**			**369**	**49643**	**4.4**	**[0.6; 8.3]**	**27.9%**
Heterogeneity: $I^2 = 89\%$, $\tau^2 = 0.0023$, $\chi^2_6 = 54.28$ ($p < 0.01$)							
**Continent = Europe**							
E. Stenager et al.	1992	Denmark	53	1444	3.7	[2.8; 4.8]	5.7%
E. Stenager et al.	1996	Denmark	50	5525	0.9	[0.7; 1.2]	6.6%
H. Ford et al.	2002	UK	2	57	3.5	[0.4; 12.1]	1.3%
S. Fredrikson et al.	2003	Sweden	90	5052	1.8	[1.4; 2.2]	6.6%
H. Hansen et al.	2004	Denmark	183	9881	1.9	[1.6; 2.1]	6.6%
C. Smestad et al.	2009	Norway	3	255	1.2	[0.2; 3.4]	5.1%
M. Sumelahti et al.	2010	Finland	15	464	3.2	[1.8; 5.3]	4.6%
A. Lalmohameda et al.	2012	Netherland	2	69	2.9	[0.4; 10.1]	1.8%
A. Manouchehrinia et al.	2014	UK	1	80	1.2	[0.0; 6.8]	3.3%
A. Zarranz et al.	2014	Spain	1	89	1.1	[0.0; 6.1]	3.6%
E. Leray et al.	2015	France	47	1569	3.0	[2.2; 4.0]	6.0%
D. Sandi et al.	2016	Hungary	4	121	3.3	[0.9; 8.2]	2.4%
H. Lunde et al.	2017	Norway	13	291	4.5	[2.4; 7.5]	3.3%
S. Kalson-Ray et al.	2017	France	47	1569	3.0	[2.2; 4.0]	6.0%
A. Murtonen et al.	2021	Finland	3	113	2.7	[0.6; 7.6]	2.6%
O. Eliasdottir et al.	2023	Sweden	0	526	0.0	[0.0; 0.7]	6.6%
**Prevalence (REM)**			**514**	**27105**	**2.1**	**[1.5; 2.8]**	**72.1%**
Heterogeneity: $I^2 = 92\%$, $\tau^2 = 0.0001$, $\chi^2_{15} = 195.58$ ($p < 0.01$)							
**Prevalence (REM)**			**883**	**76748**	**2.1**	**[1.5; 2.7]**	**100.0%**
Heterogeneity: $I^2 = 93\%$, $\tau^2 = 0.0001$, $\chi^2_{22} = 307.11$ ($p < 0.01$)							
Test for subgroup differences: $\chi^2_1 = 1.37$, df = 1 ($p = 0.24$)							

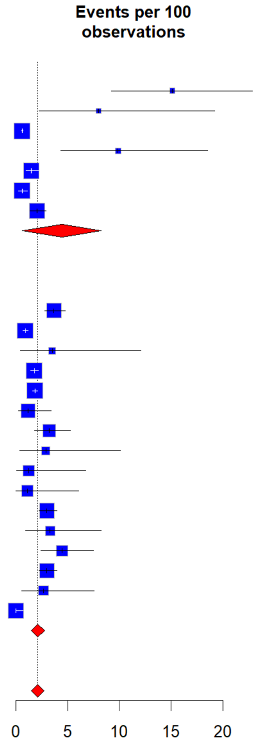

Figure S11. Funnel plot of prevalence of suicide ideation in PwMS

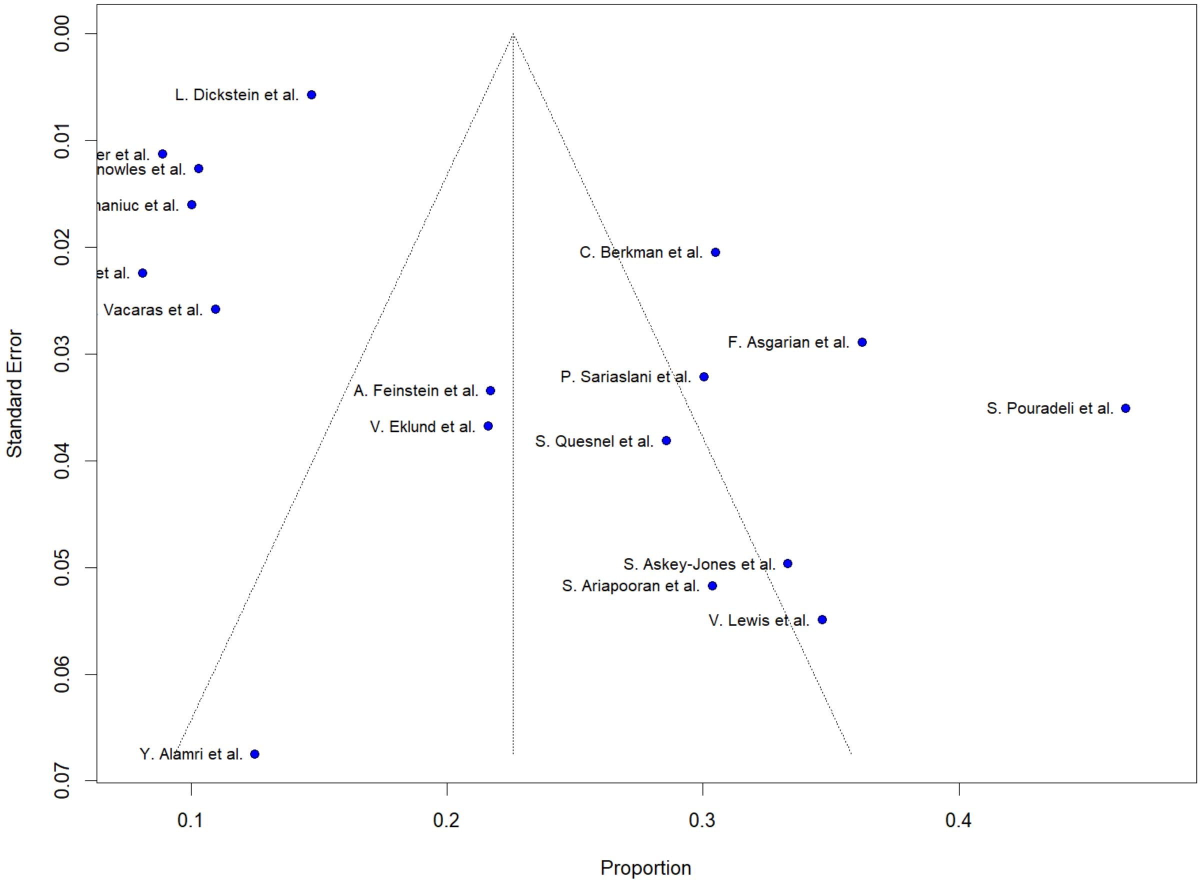

Figure S12. Funnel plot of prevalence of suicide attempt in PwMS

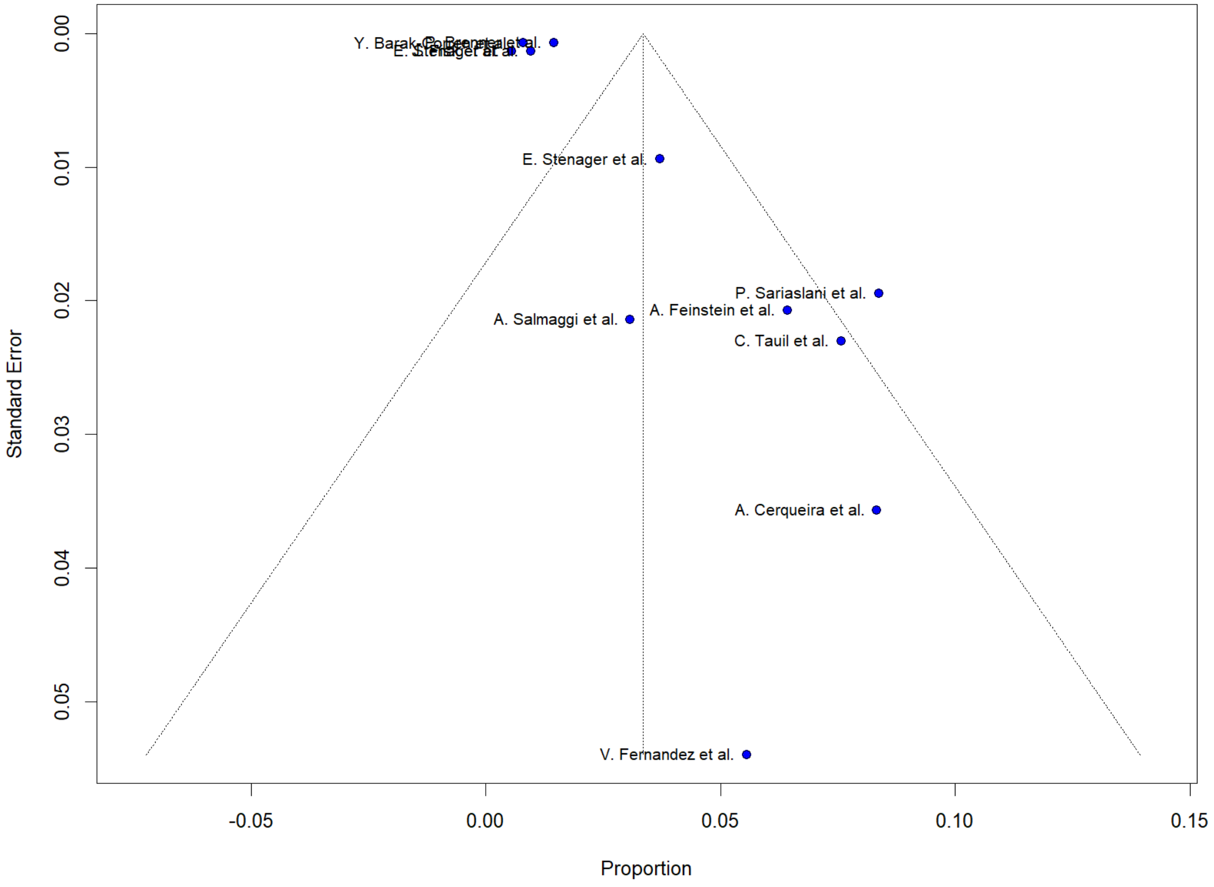

Figure S13. Funnel plot of prevalence of suicide mortality in PwMS

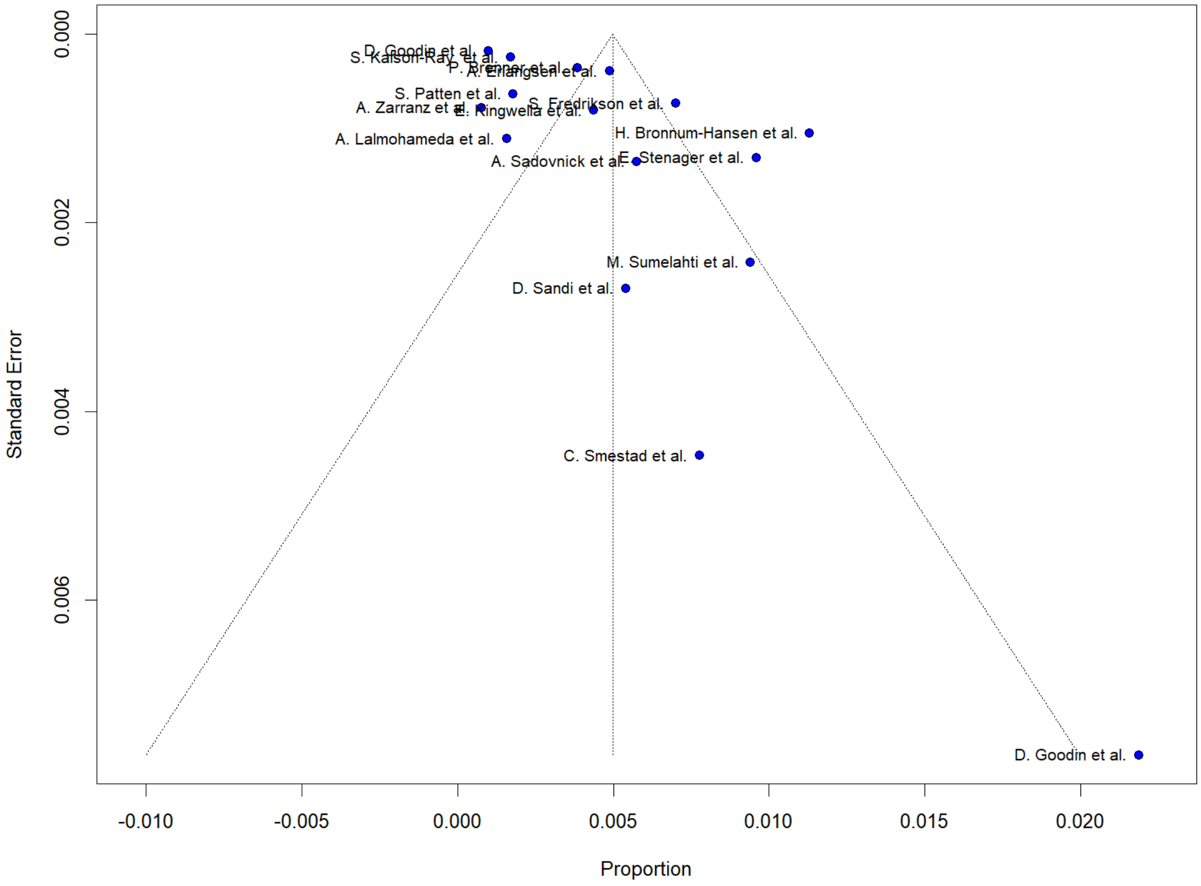

Figure S14. Funnel plot of prevalence of suicide mortality among total mortality in PwMS

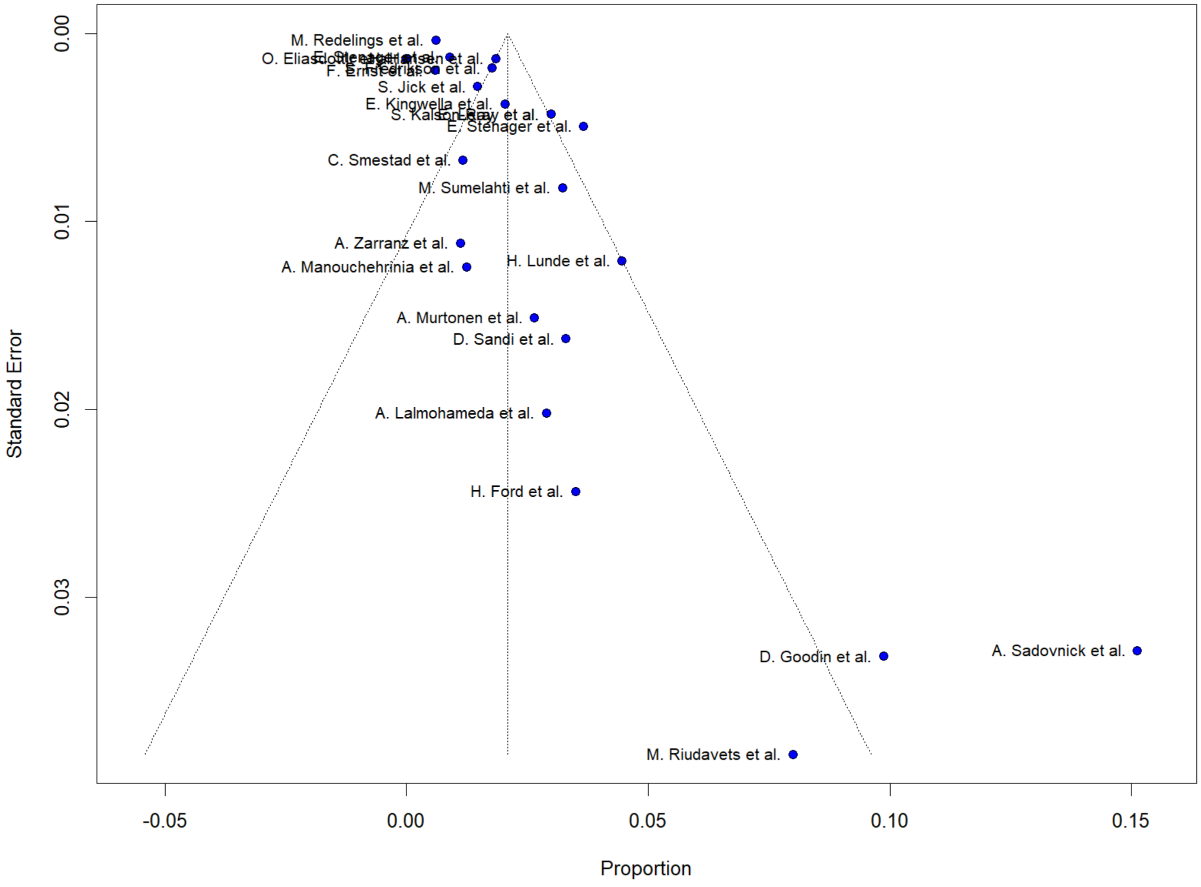

Figure S15. Funnel plot of suicide SMR

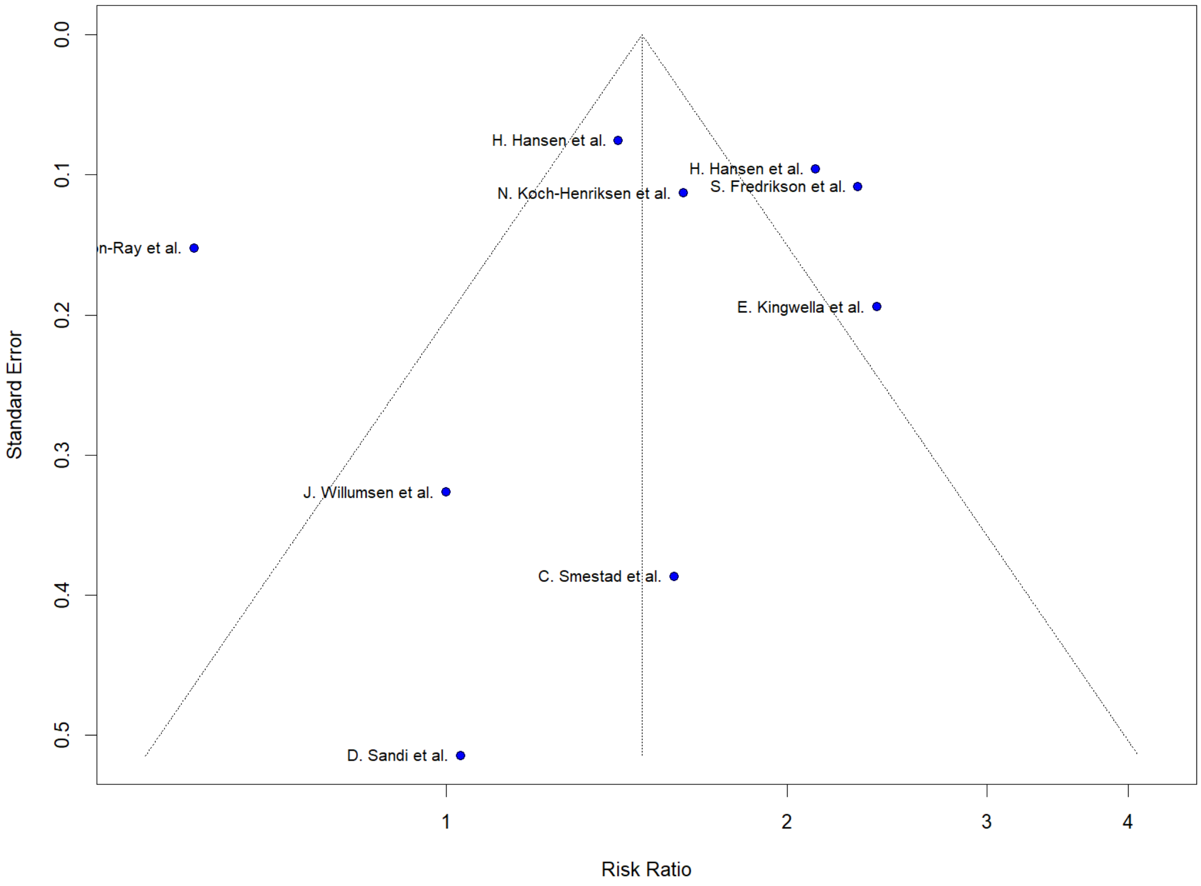

Figure S16. Sensitivity analysis of suicide SMR

Study	SMR	95%-CI	P-value	Tau2	Tau	I2
Omitting N. Koch-Henriksen et al. 1998	1.46	[1.01; 2.11]	0.04	0.2240	0.4733	90%
Omitting S. Fredrikson et al. 2003	1.39	[0.99; 1.96]	0.06	0.1888	0.4345	88%
Omitting H. Hansen et al. 2004	1.49	[1.03; 2.16]	0.03	0.2251	0.4745	90%
Omitting H. Hansen et al. 2005	1.41	[0.99; 2.00]	0.06	0.2019	0.4493	89%
Omitting C. Smestad et al. 2009	1.48	[1.04; 2.09]	0.03	0.2117	0.4601	90%
Omitting D. Sandi et al. 2016	1.52	[1.09; 2.13]	0.01	0.2001	0.4473	90%
Omitting S. Kalson-Ray et al. 2017	1.76	[1.45; 2.15]	< 0.01	0.0459	0.2143	72%
Omitting E. Kingwella et al. 2019	1.40	[1.00; 1.96]	0.05	0.1893	0.4351	89%
Omitting J. Willumsen et al. 2022	1.55	[1.10; 2.18]	0.01	0.1985	0.4456	90%
**Random effects model**	**1.49**	**[1.08; 2.05]**	**0.01**	**0.1899**	**0.4357**	**89%**

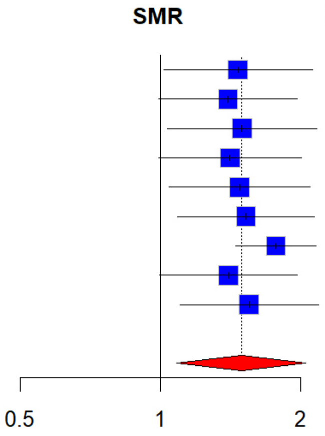

Figure S17. Funnel plot of suicide mortality OR

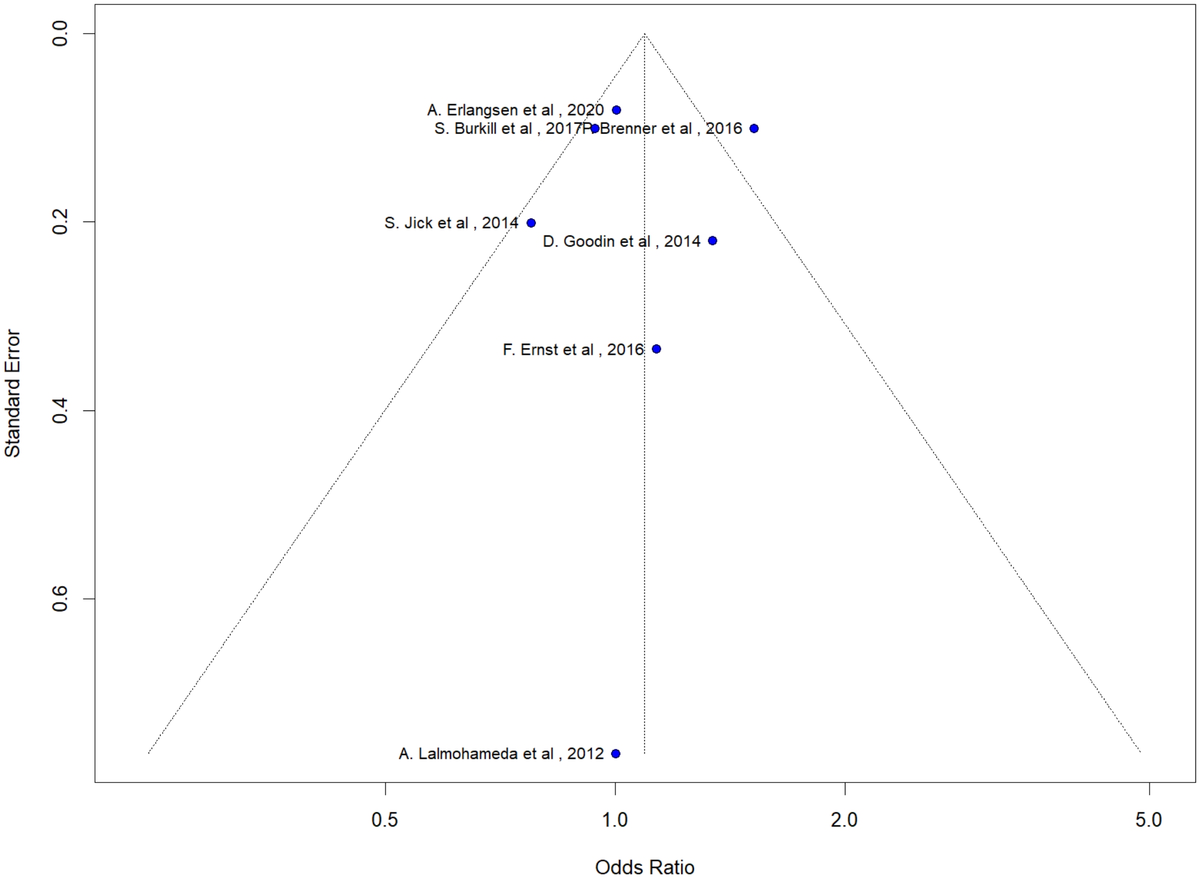

Figure S18. Sensitivity analysis of suicide mortality OR

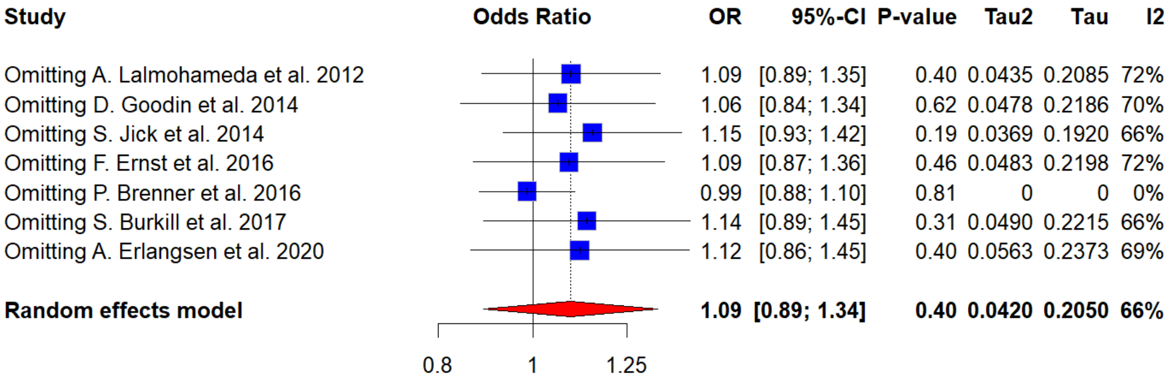

Figure S19. Pooled suicide mortality HR

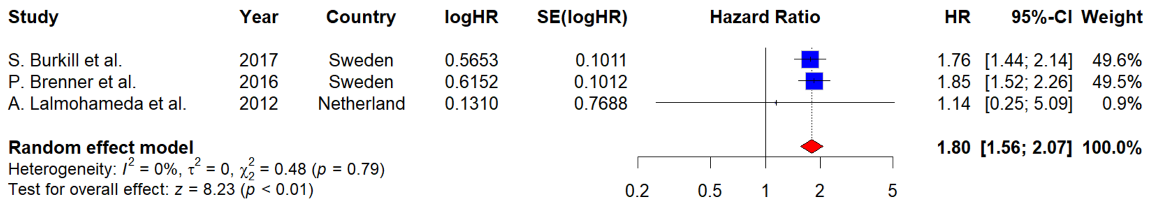

Figure S20. Funnel plot of suicide mortality HR

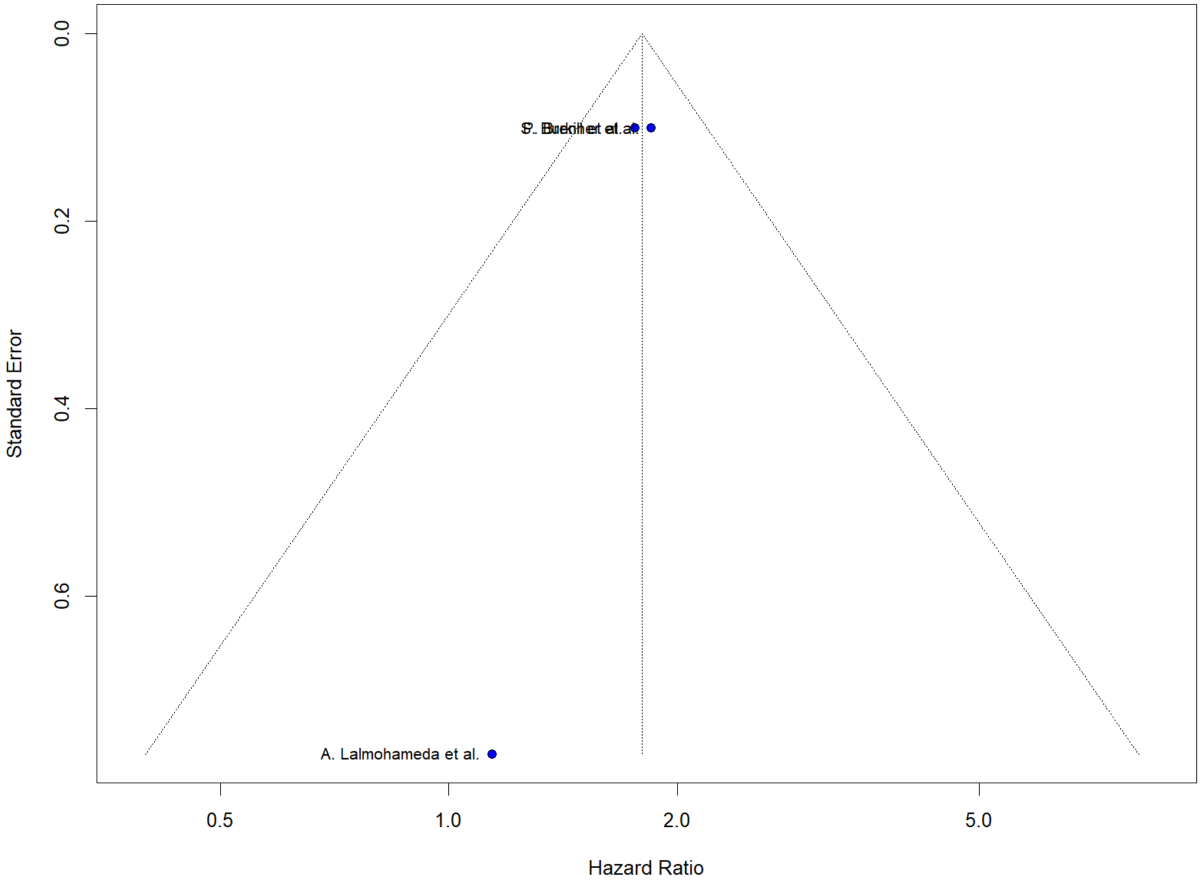

Figure S21. Sensitivity analysis of suicide mortality HR

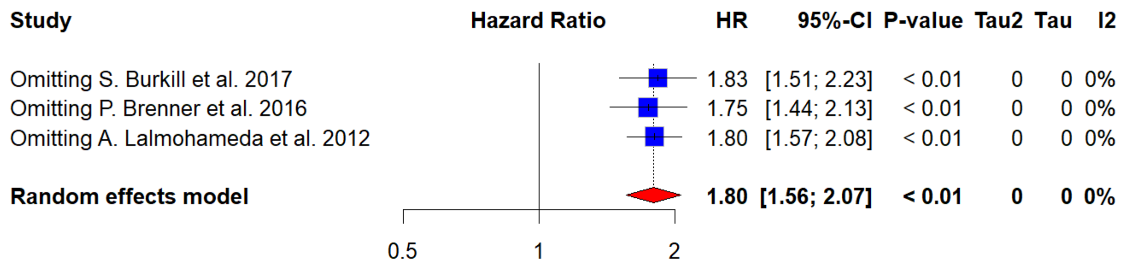

Figure S22. Pooled suicide mortality RR

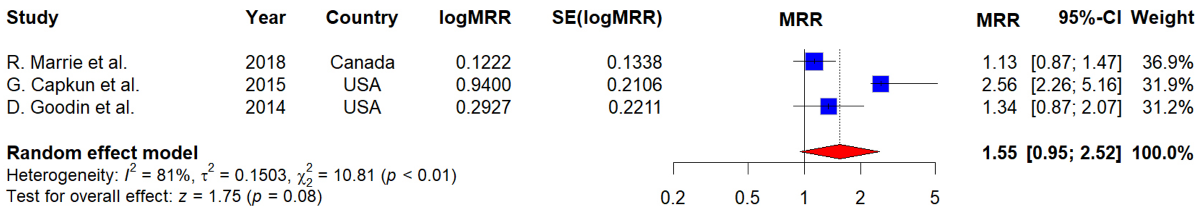

Figure S23. Funnel plot of suicide mortality RR

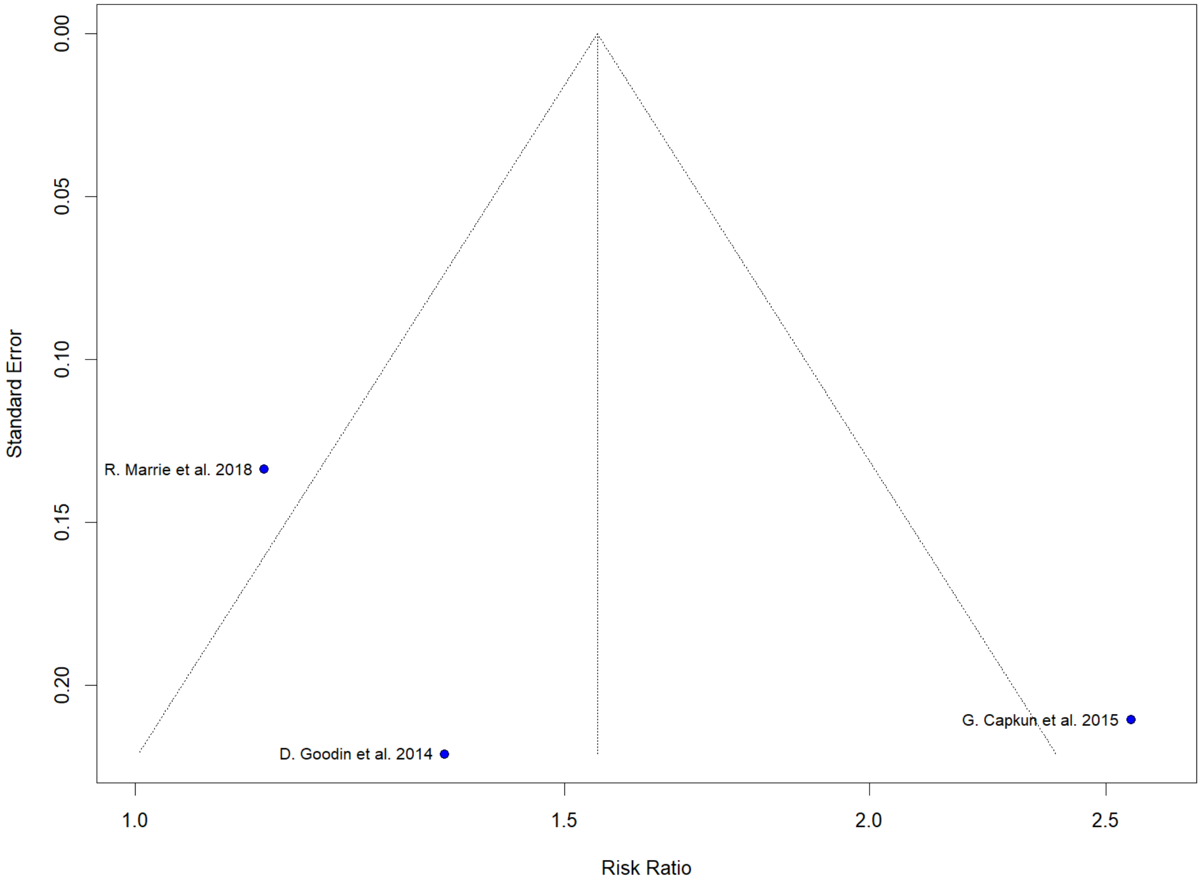

Figure S24. Sensitivity analysis of suicide mortality RR

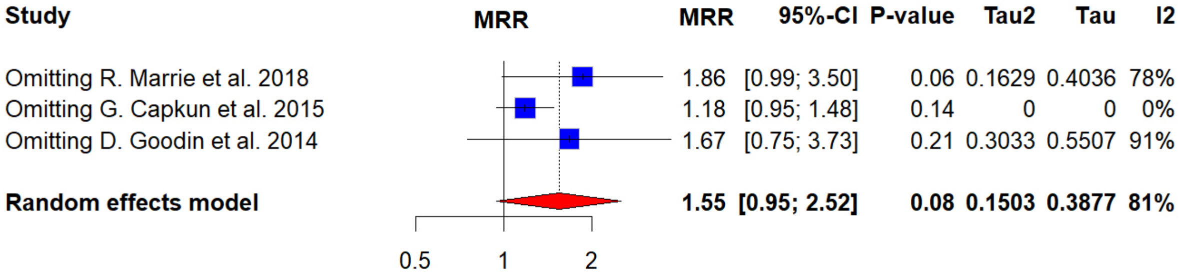

Supplement: Supplementary file 2 — Supplementary materials 2. Subgroup analyses, sensitivity analyses, and funnel plots [file BRB3-15-e70839-s002.pdf]
