# Supplementary material for: Suicide Ideation, Attempts, and Mortality in Multiple Sclerosis: A Systematic Review and Meta‐Analysis
Source: Brain Behav. 2025 Sep 9;15(9):e70839. doi: 10.1002/brb3.70839 (PMC12417961; doi:10.1002/brb3.70839)
Supplement: Supplementary file 3 — Supplementary materials 3. Comparison of Study Characteristics between OR and SMR Studies [file BRB3-15-e70839-s003.docx]

Table S2. Comparison of Study Characteristics between OR and SMR Studies.

| Study  (Author et al., Year)  [Ref] | Effect Size Reported  (OR or SMR) | Design  (Case-control or Cohort) | Sample Size | Follow-up Duration (years) | Adjustment of Variables |
| --- | --- | --- | --- | --- | --- |
| N. Koch-Henriksen et al., 1998  (1) | SMR | Cohort | 6068 | 42 | Sex  Cause of death |
| S. Fredrikson et al., 2003  (2) | SMR | Cohort | 12834 | 27 | Sex  Age  MS duration (years since diagnosed) |
| H. Hansen et al., 2004  (3) | SMR | Cohort | 9881 | 47 | Sex  Age  10-year calendar Period  Cause of death |
| H. Hansen et al., 2005  (4) | SMR | Cohort | 10174 | 46 | Sex  MS duration (years since diagnosis) |
| C. Smestad et al., 2009  (5) | SMR | Cohort | 386 | 49 | Sex  Age  10-year calendar period |
| D. Sandi et al., 2016  (6) | SMR | Cohort | 740 | 20 | Sex  Age  Calendar period |
| S. Kalson-Ray et al., 2017  (7) | SMR | Cohort | 27603 | 7 | Sex  Age  Calendar period |
| E. Kingwella et al., 2019  (8) | SMR | Cohort | 6629 | 28 | Sex  Age  5-year calendar period |
| J. Willumsen et al., 2022  (9) | SMR | Cohort | 964 | 71 | Sex  Age  10-year calendar period |
| 1. Lalmohamed et al., 2012   (10) | OR | Case-control | 1270 | NR | NR |
| D. Goodin et al., 2014  (11) | OR | Case-control | 30402 | NR | NR |
| S. Jick et al., 2014  (12) | OR | Case-control | 1822 | NR | NR |
| F. Ernst et al., 2014  (13) | OR | Case-control | 1518 | NR | NR |
| P. Brenner et al.,2016  (14) | OR | Case-control | 29617 | NR | NR |
| S. Burkill et al., 2017  (15) | OR | Case-control | 9563 | NR | NR |
| 1. Erlangsen et al., 2020   (16) | OR | Case-control | 31136 | NR | NR |

1. Koch-Henriksen N, Brønnum-Hansen H, Stenager E. Underlying cause of death in Danish patients with multiple sclerosis: results from the Danish Multiple Sclerosis Registry. Journal of Neurology, Neurosurgery & Psychiatry. 1998;65(1):56-9.

2. Fredrikson S, Cheng Q, Jiang GX, Wasserman D. Elevated suicide risk among patients with multiple sclerosis in Sweden. Neuroepidemiology. 2003;22(2):146-52.

3. Brønnum‐Hansen H, Koch‐Henriksen N, Stenager E. Trends in survival and cause of death in Danish patients with multiple sclerosis. Brain. 2004;127(4):844-50.

4. Bronnum-Hansen H, Stenager E, Stenager EN, Koch-Henriksen N. Suicide among Danes with multiple sclerosis. JOURNAL OF NEUROLOGY NEUROSURGERY AND PSYCHIATRY. 2005;76(10):1457-9.

5. Smestad C, Sandvik L, Celius EG. Excess mortality and cause of death in a cohort of Norwegian multiple sclerosis patients. Multiple Sclerosis. 2009;15(11):1263-70.

6. Sandi D, Zsiros V, Füvesi J, Kincses ZT, Fricska-Nagy Z, Lencsés G, et al. Mortality in Hungarian patients with multiple sclerosis between 1993 and 2013. Journal of the Neurological Sciences. 2016;367:329-32.

7. Kalson-Ray S, Edan G, Leray E. An excessive risk of suicide may no longer be a reality for multiple sclerosis patients. Multiple Sclerosis. 2017;23(6):864-71.

8. Kingwell E, Zhu F, Evans C, Duggan T, Oger J, Tremlett H. Causes that Contribute to the Excess Mortality Risk in Multiple Sclerosis: A Population-Based Study. Neuroepidemiology. 2020;54(2):131-9.

9. Willumsen JS, Grytten N, Aarseth J, Myklebust TÅ, Myhr K-M, Midgard R. Mortality and cause of death in multiple sclerosis in western Norway 1950–2021: a registry-based linkage study. Journal of Neurology, Neurosurgery & Psychiatry. 2022;93(11):1154-61.

10. Lalmohamed A, Bazelier MT, Van Staa TP, Uitdehaag BMJ, Leufkens HGM, De Boer A, et al. Causes of death in patients with multiple sclerosis and matched referent subjects: A population-based cohort study. European Journal of Neurology. 2012;19(7):1007-14.

11. Goodin DS, Corwin M, Kaufman D, Golub H, Reshef S, Rametta MJ, et al. Causes of death among commercially insured multiple sclerosis patients in the United States. PLoS ONE. 2014;9(8).

12. Jick S, Li L, Falcone G, Vassilev Z, Wallander M-A. Mortality of patients with multiple sclerosis: a cohort study in UK primary care. Journal of neurology. 2014;261:1508-17.

13. Ernst FR, Pocoski J, Cutter G, Kaufman DW, Pleimes D. Analysis of diagnoses associated with multiple sclerosis–related in-hospital mortality using the Premier Hospital Database. International Journal of MS Care. 2016;18(3):154-61.

14. Brenner P, Burkill S, Jokinen J, Hillert J, Bahmanyar S, Montgomery S. Multiple sclerosis and risk of attempted and completed suicide–a cohort study. European journal of neurology. 2016;23(8):1329-36.

15. Burkill S, Montgomery S, Hajiebrahimi M, Hillert J, Olsson T, Bahmanyar S. Mortality trends for multiple sclerosis patients in Sweden from 1968 to 2012. Neurology. 2017;89(6):555-62.

16. Erlangsen A, Stenager E, Conwell Y, Andersen PK, Hawton K, Benros ME, et al. Association between Neurological Disorders and Death by Suicide in Denmark. JAMA - Journal of the American Medical Association. 2020;323(5):444-54.
